# Intracoronary Delivery of Porcine Cardiac Progenitor Cells Overexpressing IGF-1 and HGF in a Pig Model of Sub-Acute Myocardial Infarction

**DOI:** 10.3390/cells10102571

**Published:** 2021-09-28

**Authors:** Cristina Prat-Vidal, Verónica Crisóstomo, Isabel Moscoso, Claudia Báez-Díaz, Virginia Blanco-Blázquez, Guadalupe Gómez-Mauricio, Guillermo Albericio, Susana Aguilar, María-Eugenia Fernández-Santos, Francisco Fernández-Avilés, Francisco M. Sánchez-Margallo, Antoni Bayes-Genis, Antonio Bernad

**Affiliations:** 1 ICREC Research Program, Germans Trias i Pujol Health Science Research Institute, Can Ruti Campus, Heart Institute (iCor), Germans Trias i Pujol University Hospital, 08916 Badalona, Spain; cpratvidal@gmail.com (C.P.-V.); abayesgenis@gmail.com (A.B.-G.); 2 CIBERCV, Instituto de Salud Carlos III, 28029 Madrid, Spain; crisosto@ccmijesususon.com (V.C.); imosgal@gmail.com (I.M.); cbaez@ccmijesususon.com (C.B.-D.); vblanco@ccmijesususon.com (V.B.-B.); mariuge@fibhgm.org (M.-E.F.-S.); francisco.fernandezaviles@salud.madrid.org (F.F.-A.); msanchez@ccmijesususon.com (F.M.S.-M.); 3 Institut d’Investigació Biomèdica de Bellvitge-IDIBELL, 08908 L’Hospitalet de Llobregat, Spain; 4 Jesús Usón Minimally Invasive Surgery Center, 10071 Cáceres, Spain; lupegmauricio@hotmail.com; 5 Cardiology Group, Center for Research in Molecular Medicine and Chronic Diseases (CIMUS), Universidade de Santiago de Compostela and Health Research Institute, University Clinical Hospital of Santiago de Compostela, 15782 Santiago de Compostela, Spain; 6 Immunology and Oncology Department, National Center for Biotechnology, 28049 Madrid, Spain; galbericio@cnb.csic.es (G.A.); saguilar@cbm.csic.es (S.A.); 7 Servicio de Cardiología, Hospital General Universitario Gregorio Marañón, Laboratorio Investigación Traslacional en Cardiología (LITC), Unidad de Producción Celular-GMP (UPC-GMP), Instituto de Investigación Sanitaria Gregorio Marañón (IiSGM), TERCEL, 28007 Madrid, Spain; 8 Departamento de Medicina, Facultad de Medicina, Universidad Complutense de Madrid (UCM), 28040 Madrid, Spain; 9 Cardiology Service, Germans Trias i Pujol University Hospital, 08916 Badalona, Spain; 10 Department of Medicine, Universitat Autònoma de Barcelona, 08193 Bellaterra, Spain

**Keywords:** cardiac progenitor cell, IGF-1, HGF, lentiviral vectors, decellularized extracellular matrix, porcine large animal model, myocardial infarction

## Abstract

Human cardiac progenitor cells (hCPC) are considered a good candidate in cell therapy for ischemic heart disease, demonstrating capacity to improve functional recovery after myocardial infarction (MI), both in small and large preclinical animal models. However, improvements are required in terms of cell engraftment and efficacy. Based on previously published reports, insulin-growth factor 1 (IGF-1) and hepatocyte growth factor (HGF) have demonstrated substantial cardioprotective, repair and regeneration activities, so they are good candidates to be evaluated in large animal model of MI. We have validated porcine cardiac progenitor cells (pCPC) and lentiviral vectors to overexpress IGF-1 (co-expressing eGFP) and HGF (co-expressing mCherry). pCPC were transduced and IGF1-eGFP^pos^ and HGF-mCherry^pos^ populations were purified by cell sorting and further expanded. Overexpression of IGF-1 has a limited impact on pCPC expression profile, whereas results indicated that pCPC-HGF-mCherry cultures could be counter selecting high expresser cells. In addition, pCPC-IGF1-eGFP showed a higher cardiogenic response, evaluated in co-cultures with decellularized extracellular matrix, compared with native pCPC or pCPC-HGF-mCherry. In vivo intracoronary co-administration of pCPC-IGF1-eGFP and pCPC-HFG-mCherry (1:1; 40 × 10^6^/animal), one week after the induction of an MI model in swine, revealed no significant improvement in cardiac function.

## 1. Introduction

The mammalian heart was believed to be a terminally differentiated organ in past decades, with no intrinsic capacity to regenerate after myocardial damage. Nevertheless, in recent times, evidences have accumulated to change this dogma. Compelling reported data confirm that the mammalian heart is in continuous slow turnover, specially concerning cardiomyocyte replenishment; in agreement, the adult heart demonstrates an intrinsic but low turnover potential contributing to normal organ homeostasis, as reviewed in Reference [1]. In human and pig hearts, myocyte proliferation is a highly regulated process, as reviewed in Reference [2], that has been demonstrated in normal young human individuals (less than 20 years old) [3] and eventually potentiated in pathologic conditions, such as myocardial infarction (MI) [4]. This suggests the existence of resident atypical progenitor cell population(s), from which new myocytes can be originated, as recently demonstrated in mammalian arteries for smooth muscle cells (SMC) [5].

Adult multipotent cardiac cells were first defined as cardiac stem cells (CSC) based on surface expression of the tyrosine kinase receptor c-kit [6]. Other cell surface markers were later proposed to describe resident subpopulations, including Sca-1, ATP-binding cassette (ABC) transporter ABCG2, or PDGFRα. This diversity of potential markers, reviewed in Reference [7], has hindered unambiguous identification and molecular definition of endogenous cardiac stem/progenitor cells (CSC/CPC), in combination with several lineage-tracing analyses that yielded non-conclusive results [8,9]. For instance, although murine c-kit-CSC expression was firstly proposed as necessary and sufficient for cardiac regeneration and repair [10], it is currently considered necessary but not sufficient to define CSC lineage [11]. In summary, evidence from several models is consistent with the involvement of CSC/CPC resident populations in cardiomyocyte turnover [8,12,13]. At present, the focus of the debate revolves around the contribution of mature cardiomyocytes by dedifferentiation/proliferation [9,14], compared with the contribution of atypical CSC/CPC. Due to their characterization, differentiation potential and function, both in homeostasis and in response to myocardial damage, CPC have been considered as an ideal candidate in cell therapy for ischemic heart disease [15].

In terms of efficacy, CSC/CPC have been reported to improve functional recovery after MI in small and large preclinical animal models. Two main heart-derived cell populations have been mainly analyzed: cardiosphere-derived cells (CDC) [16,17,18] and c-kit^+^ CPC [19,20,21]. These studies concluded that heart-derived cells have a therapeutic capacity to reduce the burden of heart disease. In the infarcted porcine heart, the deferred administration of allogeneic porcine CPC (pCPC), via the infarct-related coronary artery and at a previously defined optimal time window [20], was also demonstrated to be safe and associated with beneficial dose-dependent functional and structural improvement comparable to autologous approach [22].

Clinical trials using autologous CDC published their initial phases (CADUCEUS and TICAP), with promising results [23,24,25]. CADUCEUS confirmed a reduction of infarct scar with a simultaneous increase in viable myocardium, resulting in improved regional contractility of the infarcted area, clearly superior to previous findings using any other cell population [26]. Other authors have reported encouraging results in clinical trials conducted with autologous CSC/CPC in both ischemic and non-ischemic cardiomyopathy [25,27]. However, although promising results have been obtained with autologous cells, the long time required for cell expansion before delivery is a major drawback that conditions this therapeutic option. By contrast, an allogeneic cell treatment would allow administration early, following the ischemic event and high patient throughput [28]. To date, two small Phase I/II clinical trials using allogeneic cardiac-derived cell products for MI have been completed (ALLSTAR and CAREMI). The ALLSTAR trial (NCT01458405) aimed to determine the safety profile of allogeneic CDC administered intracoronary [29]. In the CAREMI trial (NCT02439398), patients treated with an intracoronary infusion of allogeneic CPC were followed for one year to evaluate safety and efficacy endpoints [30,31]. Although safety and feasibility of this therapy have been reported, results failed to demonstrate statistically significant efficacy. Therefore, some modifications must be incorporated to the allogeneic cells, aiming to reinforce their intrinsic therapeutic potential.

The beneficial effects of CSC/CPC were initially attributed to their capability to engraft and differentiate into various cell lineages. However, there is a growing body of evidence supporting that tissue repair is predominantly mediated by paracrine factors or extracellular vesicles secreted by CSC/CPC, with a minimal contribution of long-term engraftment and transdifferentiation of transplanted CPC [32,33,34,35]. Many reports suggest that various progenitor-secreted factors promote survival of cardiomyocytes at risk, mediate angiogenesis, and even protect against myocardial ischemia, resulting in long-lasting favorable effects despite the short survival of transplanted cells [36,37,38,39]. Other groups have aimed to improve engraftment overexpressing pro-survival factors as integrin-linked kinase (ILK), favoring also angiogenesis [40] or co-administrating accessory scaffolds to ensure local delivery and to promote cell viability, as reviewed by Reference [41]. Some studies showed that, in the adult heart, hepatocyte growth factor (HGF)/Met signaling controls several relevant aspects of heart homeostasis and prevents also oxidative stress in normal cardiomyocytes. In the injured heart, HGF plays important roles in cardioprotection by promoting pro-survival effects in cardiomyocytes, protecting against ischemia oxidative stress, promoting angiogenesis, inhibition of fibrosis, anti-inflammatory and immunomodulatory signals, and boosting regeneration through activation of CSC reviewed by References [42,43,44]. Immediate administration of adenoviral vectors carrying the HGF gene into the left ventricular (LV) wall surrounding the infarct areas of the aged rat heart induced necroptosis that seemed to facilitate aged heart repair after MI by promoting c-kit^+^ CSC proliferation and differentiation [45]; previous work in the porcine model also described activation of pro-survival pathways by HGF, inducing cardiomyocyte proliferation, and improving heart perfusion and cardiac function in pigs with chronic MI [46].

Similarly, insulin-like growth factor-1 (IGF-1), another cardioprotective growth factor, protects primary cardiomyocytes from apoptosis induced by hypoxia and serum deprivation [47], playing also a significant role in cardiomyocyte differentiation [48]. IGF-1 deficiency is associated with an increased risk of cardiovascular disease, whereas cardiac activation of IGF-1 receptor (IGF-1R) protects from the detrimental effects of MI, as reviewed in Reference [49]. In addition, bone marrow-derived mesenchymal stem/progenitor cells (BM-MSC) and CPC exosomes were compared, demonstrating an enhanced cardioprotection by CPC-exosomes associated with an incremented secretion of IGF-1 and receptor activation [36]. Concerning cell engineering with IGF-1, lentiviral overexpression of IGF-1 in BM-MSC enhanced viability, migration, anti-apoptosis, and protective effects on cardiomyocytes [50]. In an MI rat model, IGF-1-adipocyte-derived mesenchymal stem cells improved LV ejection fraction (LVEF) and cardiac contractility index but did not reduce infarct scar size when compared to the control group [51]. Similarly, the administration of microencapsulated IGF-1 in a relevant ischemia-reperfusion MI porcine model improved cardiac function and significantly decreased fibrosis in treated animals compared to controls [52].

Although the effects of administering independently different cell types or growth factors have been described [53,54], few studies have examined the combined effect of IGF-1 and HGF [55]. Interestingly, direct co-administration has been evaluated for potential treatment of acute MI both in the rat [56] and the porcine model [57]. CPC express both HGF and IGF-1, but at low levels [37], so we have evaluated here whether their equilibrated overexpression in pCPC could benefit their therapeutic potential in a large animal model of sub-acute MI, after intracoronary transplantation.

## 2. Materials and Methods

### 2.1. Cell Culture

Porcine CPC (pCPC) were isolated from small biopsies of porcine adult atria, as previously described for human cells [58,59]. Cells were expanded in Dulbecco’s modified Eagle’s medium (DMEM)/F12 medium supplemented with 10% ES cell-qualified fetal bovine serum (FBS), 100 U/mL penicillin G, 0.1 mg/mL streptomycin, 2 mM L-glutamine, 10 ng/mL gentamicin, 2.25 µg/mL fungizone, 1× ITS (all from Gibco), 0.005 U/mL hEPO (Sigma-Aldrich, St. Louis, MO, USA), 10 ng/mL FGF2 (ThermoFisher, Waltham, MA, USA), 20 µg/mL EGF (Peprotech, Madrid, Spain), and 50 µM 2-mercaptoethanol (Sigma-Aldrich) under standard culture conditions.

HEK293T (HEK293FT, Invitrogen, Waltham, MA, USA) were cultured under standard conditions in DMEM (Lonza, Basel, Switzerland) supplemented with 1% Glutamax (Invitrogen), 10 mg/mL antibiotics (penicillin/streptomycin), and 10% FBS (Sigma-Aldrich).

When indicated, porcine MSC (paMSC), human CPC (hCPC), human BM-MSC, or human dermal fibroblasts (HDF) were used for comparative analysis. paMSC were isolated from subcutaneous adipose tissue and expanded as previously described [60]. hCPC were previously purified by c-kit immunoselection and further expanded as reported [58,59]. BM-MSC were cultured in DMEM-low glucose (Sigma-Aldrich) supplemented with 10% FBS (Sigma-Aldrich), 2 mM glutamine (Lonza), 100 IU/mL penicillin, and 1000 U/mL streptomycin (Lonza). Finally, HDF were purchased from American Type Culture Collection (CRL-2097; Manassas, VA, USA) and maintained in DMEM (Sigma) supplemented with 10% FBS, 2 mM glutamine (Lonza), 100 U/mL penicillin and 1000 IU/mL streptomycin (Lonza).

### 2.2. Karyotyping and Cell Proliferation Studies

To evaluate eventual chromosomal abnormalities, pCPC were incubated with 10 μg/mL colcemid for 4 h at 37 °C, and metaphase cells were prepared by standard cytogenetic methods. Q-FISH was carried out using a FITC-conjugated LL(CCCTAA)3 peptide nucleic acid telomeric probe (Eurogentec), as previously reported [61].

pCPC growth in vitro was tracked by cell number count and population doubling level (PDL) measurement. Population doubling (PD) gained at each passage was calculated with the formula:

PD_(n/(n−1))_ = (log (N_n_/N_n−1_))/log 2

(n: passage; N: cell number). PDL is the sum of PDs.

### 2.3. Flow Cytometry

Phenotypic analysis was carried out by FACS (fluorescence-activated cell system). Specifically, pCPC were immunostained by using specific monoclonal antibodies c-kit porcine-PE (DakoCytomation, Carpinteria, CA, USA), CD34-PE, CD45-FITC, CD90-FITC, and CD166-PE (BD Pharmingen, San Diego, CA, USA). Mouse IgG1-FITC (Caltag Medsystems, Buckingham, UK), mouse IgG2a-FITC, and rat IgG1-FITC (Abcam, Cambridge, UK) were used as isotype controls (Table S1). Quantitative analyses were performed using a flow cytometer FACS Scan (BD Biosciences, Franklin Lakes, NC, USA). Additional antibodies used for comparative expression analysis between pCPC and hCPC are also listed in Table S1.

Stably transduced pCPC populations with different lentiviral vectors were sorted by eGFP or mCherry fluorescence also with the FACS technique (flow cytometer FACS Aria; BD Biosciences).

### 2.4. Cardiac In Vitro Differentiation of pCPC

Cardiac differentiation was promoted by seeding pCPC at 2 × 10^4^ cells/cm^2^ in culture medium (α-MEM supplemented with 10% FBS, 1% penicillin/streptomycin, and 1% l-glutamine) and replacing medium after 24 h with culture medium supplemented with 10 nM dexamethasone. At 0, 3, 7, 10, and 14 days, samples were collected for gene expression analysis.

### 2.5. Quantitative Real-Time PCR

Total RNA was extracted from different CPC populations using TRI-Reagent^®^ (Sigma-Aldrich, St. Louis, MO, USA) following manufacturer’s instructions. RNA concentration and purity was determined using a Nano-Drop spectrophotometer (Thermo Scientific, Waltham, MA, USA). cDNA was synthesized from 1 µg of total RNA using random primers (Invitrogen) and SuperScript^®^ III Reverse Transcriptase (Invitrogen) according to manufacturer´s protocol. Primer sequences are listed in Table S2 and were designed using Web program of the National Center for Biotechnology Information (NCBI). Quantitative real-time PCR (RT-qPCR) was performed using SYBR Green (Applied Biosystems, Waltham, MA, USA) in a Mastercycler^®^ ep realplex (Eppendorf, Madrid, Spain). RT-qPCR data were quantified by the 2^−ΔΔCt^ method, using ACTB, GUSB, or GAPDH as housekeeping genes (indicated in each analysis).

### 2.6. RNA-Seq Analysis

RNA was isolated, as previously indicated, from the different isolates: pCPC (pCSC01, pCSC03, and pCSC05), hCPC (S7, S9, and S11), BM-MSC (S1, S3, and S5), and HDF (S1_FB, S2_FB, and S3_FB). RNAseq libraries were obtained using the TruSeq RNA Sample Preparation v2 Kit (Illumina Inc., San Diego, CA, USA). The quality, quantity and the size distribution of the Illumina libraries were determined using the DNA-1000 Kit (Agilent Bioanalyzer, Santa Clara, CA, USA). Libraries were sequenced (single-end mode, 75 bp length) on the Genome Analyzer IIx System (Illumina Inc.) following the standard RNA sequencing protocol, with the TruSeq SBS Kit v5. Fastq files containing reads for each library were extracted and demultiplexed using the CASAVA v1.8.2 pipeline.

### 2.7. pCPC Ientiviral Transduction In Vitro

For transduction, pCPC were seeded on six-well plates (2 × 10^5^ cells per well) and allowed to attach for 24 h. Lentiviral vectors encoding IGF1-eGFP (pRRLsin18.CMV-IGF1-IRES-eGFP), HGF-mCherry (pRRLsin.CMV-HGF-IRES-mCherry), or the corresponding controls (Figure 3) were then added (MOI = 5), together with 8 µg/mL polybrene (Sigma), into expanded cells in complete culture medium and incubated for 6–12 h at 37 °C. After that, viral supernatant was replaced with fresh cell medium and cells returned to 37 °C for 72 h. Cells were then trypsinized and collected, washed twice with 1× PBS, and seeded for direct IF analysis or sorted by eGFP or mCherry fluorescence. Empty vectors (EV) (pRRLsin18.CMV.IRES.eGFP and pRRLsin18.CMV.IRES.mCherry) were used as negative transduction controls. When indicated, HEK293T and paMSC were transduced in parallel as additional controls.

### 2.8. Western Blot

Cells were lysed in RIPA buffer containing a protease inhibitor cocktail (Roche, Madrid, Spain). After centrifugation, supernatants were collected and protein quantified using the DC protein assay (Biorad). Protein (30 µg/lane) was loaded on 10–12% SDS-poliacrylamide gels and transferred to PVDF membrane (iBlot^®^ Get Transfer Stacks PVDF Regular, Invitrogen) using the iBlot^®^ system (Invitrogen). Membranes were blocked and incubated O/N with primary antibodies (Table S1) diluted in 5% non-fat milk in PBS, 0.1% Tween 20 (Sigma). Next, PVDF membranes were incubated with the corresponding horseradish peroxidase (HRP)-conjugated secondary antibodies (Table S1). Finally, blots were developed with HRP SuperSignal^®^ West Pico Trial (Thermo Scientific) and visualized in an LAS3000 instrument (Fujifilm, Madrid, Spain).

### 2.9. Immunofluorescence

Transfected or transduced cells were seeded onto glass coverslips coated with poly-L-lysine (Sigma-Aldrich). After 24–48 h, cells were washed twice with PBS and fixed (15 min) in 4% paraformaldehyde at RT. After permeabilization with 0.1% Triton-X, cells were blocked with 10% goat serum and incubated O/N at 4 °C with primary antibodies in 1% goat serum. Primary antibodies used are listed in Table S1. Next, cells were washed and incubated for 45 min at RT with fluorescent-conjugated secondary antibody (Table S1). Slides were mounted in Prolong^®^ Gold Antifade with DAPI (Invitrogen) and examined under a Leica SP5 confocal laser scanning microscope (Leica Microsystems, Wetzlar, Germany).

### 2.10. Co-Culture of Engineered pCPC in Decellularized Rat Cardiac Scaffolds

To obtain the rat decellularized scaffolds, we used essentially the previously described perfusion decellularization protocol to remove the cells from the heart, while retaining the decellularized extracellular matrix (dECM) (Figure S1) [62]. In brief, rats were anesthetized with a mix of ketamine (100 mg/mL stock; 100 μL/100 g body weight) and xylazine (20 mg/mL stock; 5 μL) (both from Dechra). Rats were injected intravenously with a large dose of sodium heparin (20,000 UI/mL stock; 100 μL/100 g body weight) (Fresenius Kabi, Bad Homburg, Germany), and the bioreactor was started while the heparin was taking effect. The hearts were perfused with 1% sodium dodecyl sulfate (SDS) (Sigma-Aldrich) in deionized water via antegrade flow through the ascending aorta; perfusion was stopped based on experience (the heart cleared to white after 13–24 h) and the hearts were then rinsed extensively with PBS containing 100 U/mL penicillin G and 100 g/mL streptomycin (Sigma-Aldrich). LV-dECM scaffolds were prepared, and confirmation of cell removal and retention of parenchymal and vascular structures was carried out as described [63]. Schematic summaries of both LV-dECM obtaining and co-culture strategy are shown in Figure S1a,b.

To evaluate the impact of co-culture of cardiac LV-dECM on pCPC differentiation, we seeded pCPC (5 × 10^4^ cells) overexpressing IGF-1 (pCPC-IGF1-eGFP) compared with pCPC overexpressing HGF (pCPC-HGF-mCherry) on 6-well plates (9.5 cm^2^) with LV-dECM. DMEM/F12 medium supplemented with 10% ES cell-qualified FBS, 100 U/mL penicillin G, 0.1 mg/mL streptomycin, 2 mM L-glutamine, 10 ng/mL gentamicin, 2.25 µg/mL fungizone, 1× ITS (all from Gibco), 0.005 U/mL hEPO (Sigma-Aldrich), 10 ng/mL FGF2 (Biosource), 20 µg/mL EGF (Peprotech), and 50 µM 2-mercaptoethanol (Sigma-Aldrich) under standard culture conditions were used; co-cultures were maintained up to 21 days, with cell culture medium replacement every 2–3 days. For internal comparisons, pCPC-IGF1-eGFP and pCPC-HGF-mCherry cells were also expanded in conventional 2D culture on gelatin-pretreated (0.1%) (Sigma-Aldrich) culture plates. After co-culture, cells were harvested, and the eventual differential effect of LV-dECM on the differentiation of pCPC-IGF1-eGFP and pCPC-HGF-mCherry cells, in comparison with the conventional 2D cultures, were evaluated by RT-qPCR.

### 2.11. Transplantation of Engineered pCPC in Swine Myocardial Infarct Model

All procedures were performed under general anesthesia: animals were premedicated by intramuscular diazepam (0.2 mg/Kg) and ketamine (Ketamidor 100 mg/mL, Richter Pharma AG, Wels, Austria), (15 mg/Kg), induction was achieved with intravenous 1% propofol (Propofol-Lipuro; Bbraun, Melsungen, Germany) (3 mg/kg), and endotracheal intubation with cuffed tubes performed. Animals were connected to a semi-closed circular circuit attached to a ventilator (Maquet Flow i), and maintenance was performed with sevoflurane in oxygen (1.8–2% inspiratory fraction). Ventilation was controlled with a tidal volume of 8–10 mL/kg at an adjusted rate to obtain normocapnia values (35–40 mmHg of CO_2_). Lidocaine (Lidocaína 2% Braun, B/Braun) was administered continuously at a rate of 1 mg/kg/h for antiarrhythmic prophylaxis. Anesthetic monitoring included cardiovascular and hemodynamic parameters, such as: heart rate, electrocardiography, pulse-oximetry, and invasive arterial blood pressure.

As post-operative analgesia, swine received 0.3 mg/12 h intramuscular buprenorphine for one day, along with a 50 μg/h transdermal fentanyl patch.

Twenty female Large White pigs were subjected to infarct induction as described elsewhere [52]. Briefly, a 7Fr. introducer sheath was percutaneously inserted into the femoral artery, and a 6Fr. hockey stick was advanced to the origin of the left anterior descending coronary artery (LAD). A balloon catheter (typically, 2.5 mm to 3.5 mm in diameter; Ryujin plus PTCA dilatation catheter, Terumo, Inc., Tokyo, Japan) was inserted over a 0.014 guidewire and inflated to temporarily occlude the LAD immediately below its first diagonal branch. Correct occlusion was documented via the manual injection of contrast agent and maintained for 90 min. Upon deflation, LAD patency was assessed with another manual contrast injection, and the animal was kept under observation for 60 min to treat eventual arrhythmic episodes and then recovered from anesthesia and returned to the pens for post-operative care.

Cardiac magnetic resonance (CMR) was performed one week after infarction to check compliance with the following inclusion criteria: infarct size > 10% of the left ventricle and LVEF < 40%.

CMR images were acquired one and 10 weeks after infarction, as previously described [20]. Briefly, a 1.5T system (Intera 1.5 T, Philips Medical Systems) and a 5-element cardiac coil were used for imaging. Cardiac function, including LVEF, and ventricular volumes indexed to Body Surface Area (end diastolic (LVEDVi) and end systolic (LVESVi) volumes) were measured in short axis breath hold gradient echo cine images. Delayed enhancement images in the short axis view were processed to determine infarct size (IS).

One week after CMR, animals were allocated to treatment (Group 1) or control (Group 2) using a random number generation. Cell vials were received with a code identifying one of four pCPC populations, so that both the preparation of the administered doses and the intracoronary cell injection were performed by blinded operators.

- Group 1: Treatment group, received subpopulations A + C = pCPC-HGF-mCherry + pCPC-IGF1-eGFP (20 × 10^6^ cells each subpopulation/animal at 2 × 10^6^ cells/mL, total volume 20 mL).
- Group 2: Control group, received subpopulations B + D = pCPC-mCherry + pCPC-eGFP (20 × 10^6^ cells each subpopulation/animal at 2 × 10^6^ cells/mL, total volume 20 mL).

As previously described [20], access to the LAD was obtained via a percutaneous femoral approach, and a 3Fr. microcatheter (Microferret infusion catheter, Cook Medical) advanced to the level of the previous coronary occlusion (immediately below the first diagonal branch). Injection was performed through the microcatheter at a rate of 1 mL/min during 4 min, followed by a 3 min pause to improve cell extravasation, and this injection cycle was repeated until injection was completed. A completion coronary angiogram was performed 5 min after administration to assess coronary patency. The femoral sheath was then removed and hemostasis of the puncture site achieved by manual compression.

CMR images were acquired also 10 weeks after infarction, as previously described [22]. Once the follow-up was completed and the 10 weeks CMR performed, animals were euthanized by a lethal dose of potassium chloride (1–2 mmol/kg) while under deep anesthesia. Hearts were excised and sliced into 10–15 mm thick slices, that were stained with a triphenyltetrazolium chloride (TTC) solution, in order to macroscopically assess IS.

Clinical efficacy was studied in terms of cardiac function, as determined using CMR, comparing LVEF, indexed ventricular volumes, IS, and therapeutic effect, defined as the changes seen over time in the calculated parameters (ΔLVEF, ΔLVEDVi, ΔLVESVi, ΔIS).

### 2.12. Statistical Analysis

Data are presented as mean ± sd. The significance of differences between groups was determined by Student’s *t*-test for paired experiments. Two-way ANOVA (with Bonferroni or Tukey’s post hoc test) were used for multiple group comparisons. The Wilcoxon paired samples test was used to assess intergroup differences for cardiac function parameters. Calculations were performed using the SPSS 22.0 statistical package for Windows (SPSS, Inc., Chicago, IL, USA) or Prism 6.0 (GraphPad software, San Diego, CA, USA), and values of *p* < 0.05 were considered statistically significant.

## 3. Results

### 3.1. Characterization of Adult pCPC

**Figure 1:**
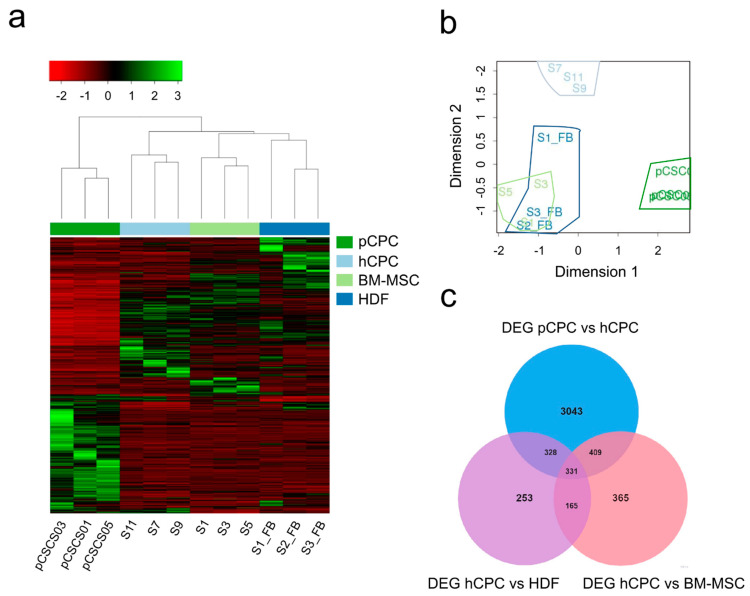
RNA−Seq analysis of pCPC compared with hCPC, BM−MSC, and HDF. (**a**,**b**) RNA−Seq experiments were carried out and analyzed using the Ilumina platform, with replicates and/or technical duplicates of all samples (see Materials and Methods section). Analysis of three pCPC isolates (pCSCS01, pCSCS03, and pCSCS05), compared with three hCPC isolates (S7, S9, and S11), three human MSC (S1, S3, and S5), and three HDF isolates (S1_FB, S2_FB, and S3_FB). (**a**) Normalized heat map analysis of expressed genes and (**b**) clustering analysis of differentially expressed genes (DEG) revealed that hCPC, BM−MSC, and HDF cell lineages are quite distant from pCPC and represent significantly differentiated clusters. (**c**) Venn diagram representing specific DEG in pCPC versus hCPC (blue color), hCPC versus BM-MSC (pink color), and hCPC versus HDF (purple color); common genes are also represented.

pCPC were isolated from porcine cardiac biopsies and expanded (passages 2–6) according to conventional procedures for hCPC. In order to get a deeper comprehension of pCPC populations, a RNA-Seq analysis was carried out (Figure 1a–c; Table S3) comparing three independent pCPC isolates with previous analyzed hCPC isolates, relative to human BM-MSC and HDF as a distant reference [37]. Globally, pCPC lineage shows a more distant expression profile with respect to the 3 human cell types (hCPC, BM-MSC, and HDF); actually, pCPC, when compared with hCPC, showed more differential expressed genes, than in comparison with BM-MSC or HDF (Figure 1b,c). However, among the genes (1003) that present the highest differences (>2 LogFC; ranging up to 15.1 LogFC) in pCPC compared with hCPC (Table S4), we did not find any of the highly preferentially CPC expressed genes that have been validated previously in hCPC [64], with the exception of IGF-1 (6.77 LogFC), KLF4 (2.89 LogFC), and F11R (2.45 LogFC).

**Figure 2:**
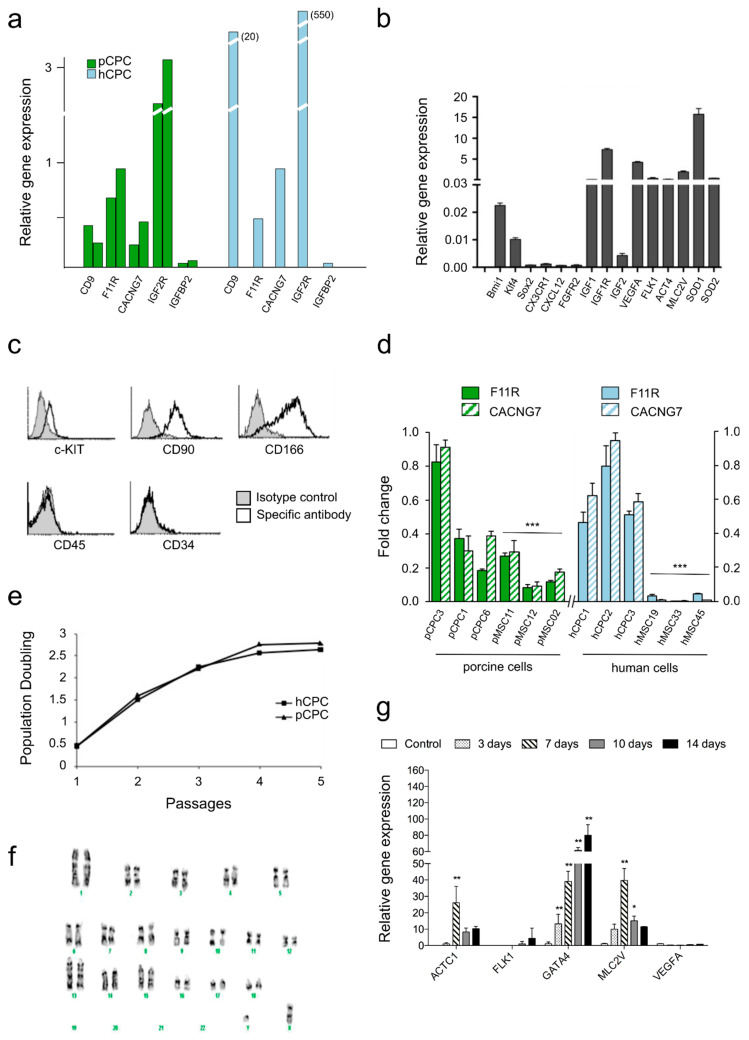
Comparative characterization of pCPC with hCPC. (**a**) Relative gene expression levels analyzed by RT−qPCR in pCPC and hCPC of five selected relevant genes, previously characterized in hCPC. (**b**) Global RT−qPCR expression analysis of pCPC. (**c**) Flow cytometry analysis of pCPC relevant surface markers. (**d**) Expression of F11R and CACNG7 in different porcine and human CPC and MSC cell isolates. ACTB was used as housekeeping gene (*n* = 3). *** *p* < 0.001 vs. pCPC3. (**e**) Representative parallel population doubling analysis in pCPC compared with hCPC. (**f**) Normal pCPC karyotype from a representative cell batch at culture passage 6. (**g**) Cardiogenic differentiation of pCPC in 2D culture during two weeks with dexamethasone (10 mM); some relevant genes were monitored at different stages of cardiogenic differentiation. ACTB was used as housekeeping gene (*n* = 3). * *p* < 0.05, ** *p* < 0.01 vs. Control.

Table 1 summarizes the genes that have been previously identified in hCPC as differentially overexpressed in comparison with BM-MSC and HDF [37,64], organized by subcellular compartments. Most of them show a reasonable comparative level of expression with the exception of GPR4 (−7.74 LogFC) and CDH5, CD9, and PTRF that are not expressed or at very low comparative levels in pCPC. To validate these findings, we compared, both in two different isolates of pCPC and hCPC, a selection of markers highly preferentially expressed in hCPC; IGFBP2 was used as a negative control being not expressed in hCPC [64]. All of them showed a compatible expression level in pCPC compared with hCPC although IGF2R and CD9 were significantly overexpressed in human cells (Figure 2a). Further RT-qPCR analysis in pCPC demonstrated significant expression of IGF-1R, VEGFA, MLC2V, and SOD1; medium level of IGF-1, FLK1, ACT4, and SOD2; and low level of Bmi1, KLF4, and IGF-2. They did not express (absent or quite weak) SOX2, CXCL12, or FGFR2 (Figure 2b). This gene expression profile was consistent with previous reported studies in hCPC [37,65]. Finally, basal gene expression of IGF-1, HGF, IGF-1R, and Met was also compared between all CPC populations (porcine and human) (Figure S2). As expected, a low expression level was found for IGF-1 and HGF both in human and pig cells, and similar levels of IGF-1R and Met receptors were detected.

Furthermore, pCPC populations were phenotypically characterized by flow cytometry, demonstrating that they were clearly positive for CD90 and CD166, moderate positive for CD117 (c-kit), and negative for CD45 and CD34 (Figure 2c), in agreement with CPC immunophenotype. A more extensive analysis of cell surface markers in pCPC compared with hCPC, as well as published markers in murine CSC populations, are included in Table S5.

The potential variability in cell surface markers expression between different pCPC batches, compared with two hCPC isolates, is shown, as well (Table S6). In this sense, we further analyzed by RT-qPCR the expression of F11R (F11 Receptor; JAM-A) and CACNG7, which have been proposed as plausible markers for hCPC. A preferential expression of both genes in several batches of pCPC compared with paMSC was confirmed, but a higher variability and less net differences in comparison with human cells (CPC versus BM-MSC) was found (Figure 2d). Substantial consistency was confirmed for pCPC versus hCPC in most surface markers evaluated. Furthermore, we corroborated that pCPC showed a quite comparable growth kinetics to hCPC (Figure 2e), and genetic stability at advanced cell passage (5–6) was also verified (Figure 2f).

Finally, preliminary functional validation of pCPC was performed, evaluating their response to cardiogenic differentiation. Figure 2g shows a continued and progressive expression level of the critical cardiogenic transcriptional factor GATA4 (days 0–14) and increased, but variable, levels of ACTC1 and MLC2V, both peaking at day 7 of differentiation treatment.

### 3.2. Generation and Characterization of pCPC with Forced IGF-1 or HGF Overexpression

**Figure 3:**
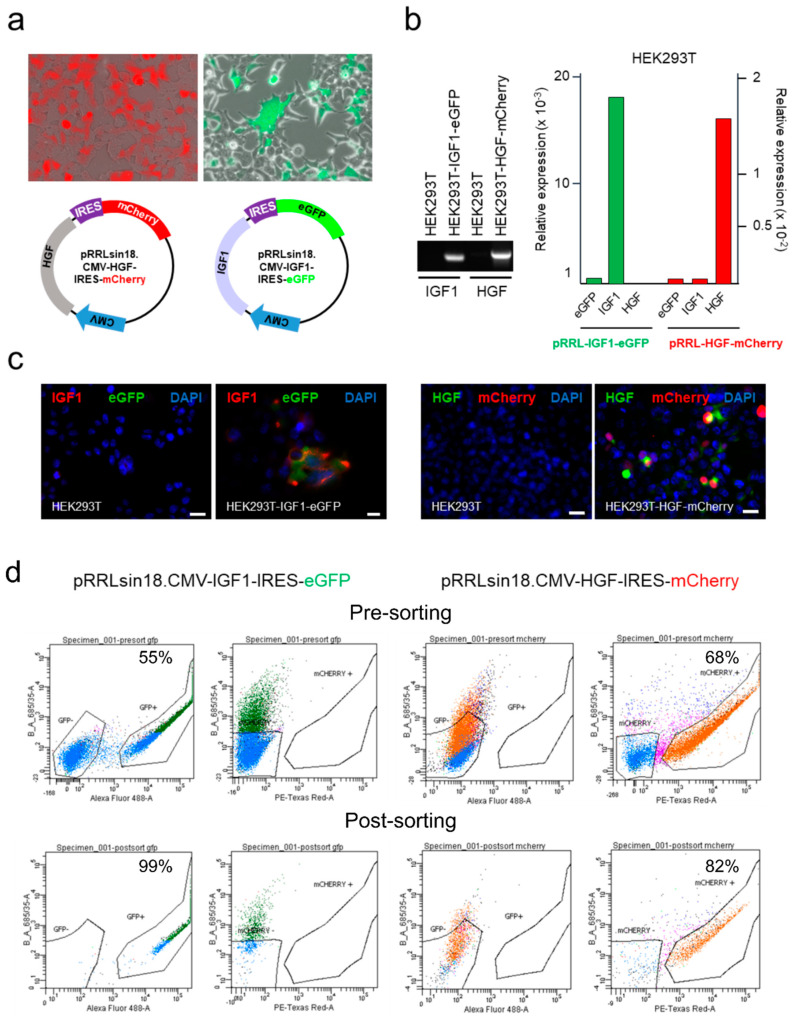
Lentiviral transduction of pCPC for IGF−1 and HGF overexpression. (**a**) Microscopy images at 200× magnification of transiently transfected HEK293T and schematic representation of the lentiviral vectors pRRLsin18.CMV-HGF-IRES-mCherry and pRRLsin18.CMV-IGF1-IRES-eGFP. (**b**) Overexpression analysis of IGF−1 and HGF by RT−qPCR in HEK293T cells transfected with IGF-1/eGFP and HGF/mCherry. Non-transfected cells were used as negative control. (**c**) Immunofluorescence (IF) analysis of transfected HEK293T co-expressing IGF1/eGFP or HGF/mCherry. Scale bar represents 20 µm. (**d**) Cytometric analysis of IGF1/eGFP and HGF/mCherry transduced pCPC confirming cell enrichment post-sorting.

Lentiviral vectors pRRLsin18.CMV-IGF1-IRES-eGFP and pRRLsin18.CMV-HGF-IRES-mCherry were generated for the combined co-expression of IGF1-eGFP and HGF-mCherry, respectively (Figure 3a), using the EV as controls [60]. Both vectors were firstly validated by transfection in HEK293T cells coupled to RT-qPCR analysis. Transfection with pRRLsin18.CMV-IGF1-IRES-eGFP or pRRLsin18.CMV-HGF-IRES-mCherry induced a very significant increment of the respective growth factor mRNA (>18 × 10^3^ fold, for IGF-1, and >5 × 10^2^ fold, for HGF) in comparison of HEK293T cells transfected with the EV; low background existed and no influence of one of the growth factor on the expression of the other was observed (Figure 3b). Immunofluorescence (IF) analysis of transfected HEK293T demonstrated that both vectors induced comparable numbers of HEK293T co-expressing IGF1-eGFP or HGF-mCherry (Figure 3c).

**Figure 4:**
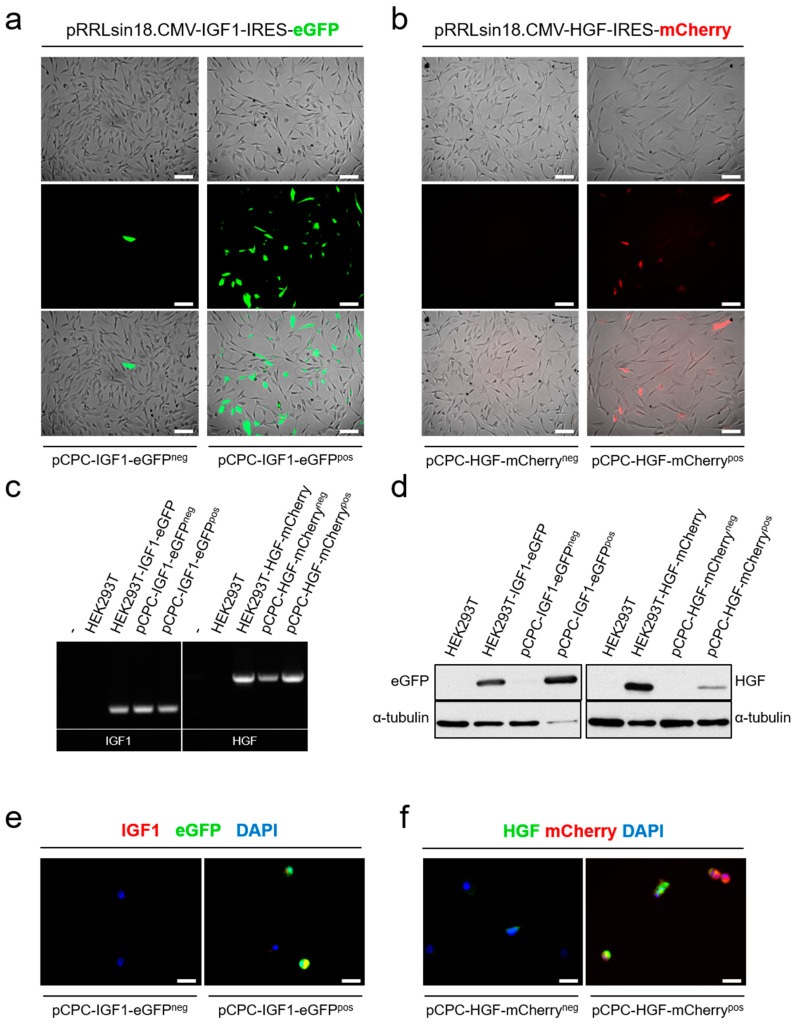
Expression analysis in pCPC IGF1/eGFP and HGF/mCherry transduced cells. (**a**) Fluorescent protein expression (eGFP) on transduced pCPC-IGF1-eGFP after cell sorting purification, compared with negative cell fraction. Scale bar represents 50 µm. (**b**) Fluorescent protein expression of mCherry on transduced pCPC-HGF-mCherry after cell sorting purification compared with negative cell fraction. Scale bar represents 50 µm. (**c**) IGF−1 and HGF expression by RT−qPCR in IGF1/eGFP and HGF/mCherry engineered cells, both in HEK293T and pCPC. (**d**) eGFP and HGF overexpression analyzed by western blot in different populations of HEK293T and pCPC. (**e**) IF analysis of IGF1/eGFP expression in pCPC-IGF1-eGFP^pos^ compared with pCPC-IGF1-eGFP^neg^ cells. (**f**) IF analysis of HGF/mCherry expression in pCPC-HGF-mCherry^pos^ compared with pCPC-HGF-mCherry^neg^ cells. Scale bar represents 20 µm.

Lentiviral particles were obtained and used to transduce pCPC cells (MOI 5); five days after transduction, pCPC-eGFP^pos^ and pCPC-mCherry^pos^ cells were analyzed by flow cytometry (55 and 68%, respectively), and positive cells were sorted (Figure 3d) and further expanded. Five days after sorting, pCPC-eGFP^pos^ and pCPC-mCherry^pos^ cells were analyzed by IF to confirm cell enrichment, by comparison with the corresponding negative population (pCPC^neg^), and the stability of fluorescent protein expression (Figure 4a,b); expression enrichment and stability seemed to be less clear in case of pCPC-mCherry^pos^ cells than pCPC-eGFP^pos^ cells. RT-qPCR analysis confirmed similar levels of IGF-1 and HGF overexpression in pCPC and HEK293T cells (Figure 4c); compared with hCPC, pCPC-eGFP^pos^ and pCPC-mCherry^pos^ cells showed no significant alterations on expression levels of IGF-1R and MET receptors (Figure S2).

Several antibodies were evaluated by western blot; signal for HGF was net, but no antibody was successfully found to confirm IGF-1 signal (Figure S3a); therefore, we used levels of eGFP expression for further quantification. Figure 4d shows a clear detection of HGF in pCPC-HGF-mCherry^pos^ cells when compared with pCPC-HGF-mCherry^neg^ cells; however, overexpression level was quite lower to that obtained in HEK293T; basal levels were very low. On the contrary, overexpression of IGF-1 in pCPC-eGFP^pos^ cells (evaluated by eGFP expression) was quite high and comparable to levels obtained in HEK293T (Figure 4d). IF analysis confirmed high levels of pCPC-IGF1-eGFP^pos^ cells co-expressing IGF-1 (Figure 4e). However, in pCPC-HGF-mCherry^pos^ cells, co-expression of HGF-mCherry was lower with a significant proportion of mCherry^pos^ HGF^neg^ (Figure 4f). Additionally, IGF-1 and HGF secretion was also verified by ELISA in the conditioned media of the corresponding pCPC transduced populations (Figure S4). Evaluation of both lentiviral vectors was also carried out in paMSC and used as an additional control of overexpression. Results indicated a comparable scenario even with a lower overexpression of HGF (Figure S3b).

### 3.3. Evaluation of the Potential Effect of IGF-1 and HGF Overexpression on pCPC Gene Expression Profile

**Figure 5:**
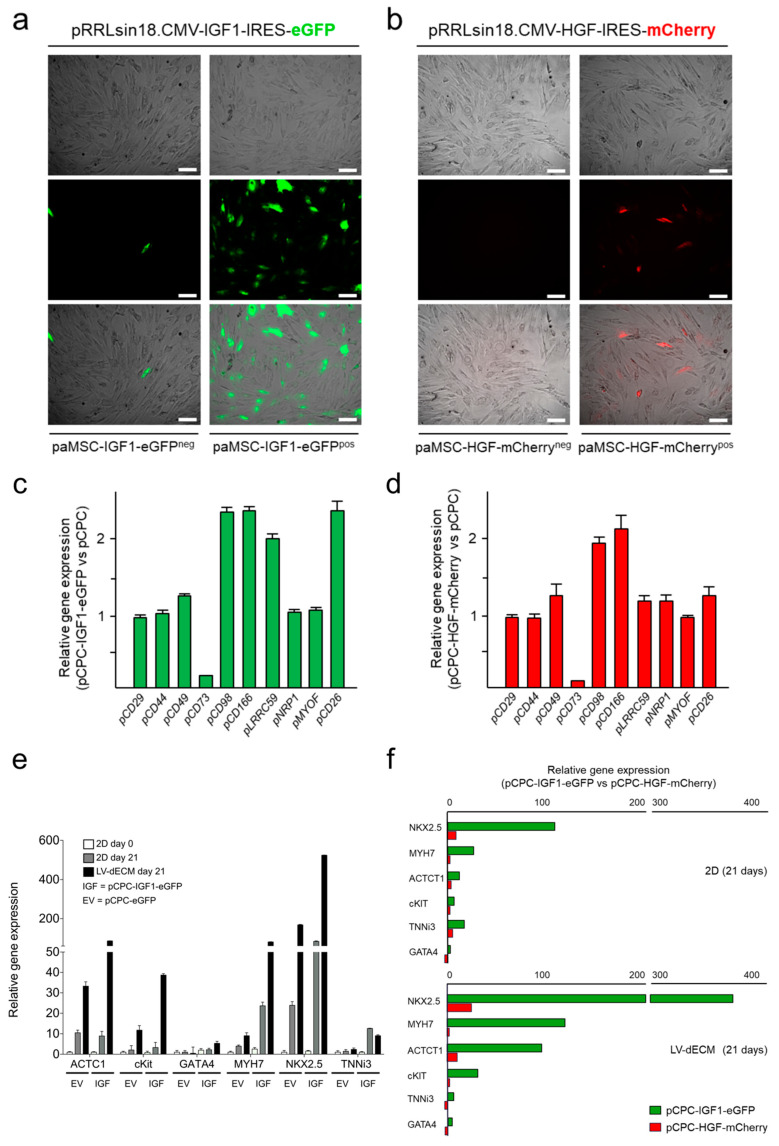
Comparative characterization of IGF1/eGFP and HGF/mCherry in both transduced paMSC and pCPC. (**a**) Fluorescent protein expression (eGFP) on transduced paMSC-IGF1-eGFP after cell sorting purification, compared with negative cell fraction. Scale bars represents 20 µm. (**b**) Fluorescent protein expression of mCherry on transduced paMSC-HGF-mCherry after cell sorting purification compared with negative cell fraction. (**c**) Relative gene expression profile of pCPC-IGF1-eGFP^pos^ cells compared with untransduced pCPC by RT-qPCR (*n* = 2). (**d**) Relative gene expression profile of pCPC-HGF-mCherry^pos^ cells compared with untransduced pCPC by RT-qPCR (*n* = 2). (**e**) Cardiogenic gene expression by RT-qPCR of pCPC-IGF1-eGFP (IGF) or pCPC-eGFP (empty vector, EV) co-cultured with decellularized rat left ventricle (LV-dECM) scaffolds for 21 days, compared with standard 2D culture (*n* = 3). (**f**) Cardiogenic gene expression by RT-qPCR of pCPC-HGF-mCherry compared with pCPC-IGF1-eGFP cells co-cultured with LV-dECM scaffolds for 21 days. Results correspond to a representative experiment out of 3 assays. GAPDH was used as housekeeping gene.

In order to evaluate whether IGF-1 or HGF overexpression could modify pCPC gene expression profile, pCPC-IGF1-eGFP^pos^ cells and pCPC-HGF-mCherry^pos^ cells were compared by RT-qPCR with untransduced cells for a panel of relevant markers. Moderate up-regulation (~2-fold) of pCD98 (Solute Carrier Family 3 Member 2; SLC3A2) and pCD166 (Activated Leukocyte Cell Adhesion Molecule; ALCAM) were found, both in pCPC-IGF1-GFP^pos^ (Figure 5a) and pCPC-HGF-mCherry^pos^ cells (Figure 5b). pCD26 (DPP4; Dipeptidyl Peptidase 4) and pLRRC59 (Leucine Rich Repeat Containing 59) appear moderately upregulated in pCPC-HGF-mCherry^pos^ compared with pCPC-IGF1-GFP^pos^ cells. Interestingly, pCD73 (5′-Nucleotidase Ecto; NT5E) was the only gene found significantly downregulated in both cell types (Figure 5a,b).

### 3.4. IGF-1 Synergizes with Decellularized Rat Cardiac Scaffolds in the Promotion of Cardiogenic Differentiation of pCPC

Next, we evaluated the behavior of engineered-pCPC after seeding on acellular rat cardiac matrix derived from LV, LV-dECM (Figure S1). First, pCPC-IGF1-eGFP and pCPC-eGFP were co-cultured with dECM during three weeks and compared with the conventional 2D culture in gelatin, as control; values for a panel of relevant cardiogenic genes were compared with the initial level of expression (day 0). Most of the analyzed genes were upregulated at day 21 compared with basal values. The analysis of a panel of relevant cardiogenic genes (day 21) showed that the expression of some genes (i.e., ACTCT1, c-KIT, and GATA4) were not modified in 2D cultures independently of the IGF-1 overexpression (EV vs. IGF). On the contrary, TNNi3 (10-fold), MYH7 (5-fold), and NKX2.5 (3-fold) were clearly upregulated when pCPC-IGF1-eGFP were used in conventional 2D culture in gelatin, compared with pCPC transduced with EV-eGFP (Figure 5e).

Finally, in comparison with 2D culture, co-culture during three weeks with dECM promoted expression of most of the genes analyzed in pCPC-IGF1-eGFP (from 3- to 370-fold); those genes showing the lowest upregulation level of expression ratio (GATA4 and TNNi3) seem not to be regulated by the co-culture with dECM (Figure 5e); no modification in IGF-1 levels was found (not shown). Co-culture of pCPC-IGF1-eGFP with dECM promoted, in most cases, the maximum up-regulation (Figure 5e). Statistical analysis of gene expression data from Figure 5e is also included as supplementary material (Table S7).

Equivalent analysis with pCPC-HGF-mCherry and pCPC-mCherry showed compatible but significantly poorer results (Figure 5f and Figure S2c,d). pCPC-mCherry cells did not show any important upregulation of the analyzed cardiogenic genes (only Nkx2.5, ACTC1, and TNNi3 showed a moderate increment), neither in conventional 2D culture nor co-cultured with dECM. Curiously, HGF was significantly up-regulated in co-culture with dECM (25-fold; Figure S2c,d).

### 3.5. In Vivo Evaluation of pCPC-IGF1-eGFP and pCPC-HGF-mCherry Co-Administration for the Treatment of Swine Myocardial Infarct

Two animals died during infarct induction due to untreatable arrhythmias, and two other animals (randomized to group 2) did not meet inclusion criteria, so that 16 animals were subjected to blinded cell therapy administration, with *n* = 9 randomized to the treatment group (animals receiving injection of pCPC-HGF-mCherry + pCPC-IGF1-eGFP; Group 1) and *n* = 7 to the control group (animals receiving injection of pCPC-mCherry + pCPC-eGFP; Group 2). Injection was successfully completed in all cases, in absence of any complications. Coronary patency was preserved in all animals after injection, and no major adverse cardiovascular events were observed in this study.

**Figure 6:**
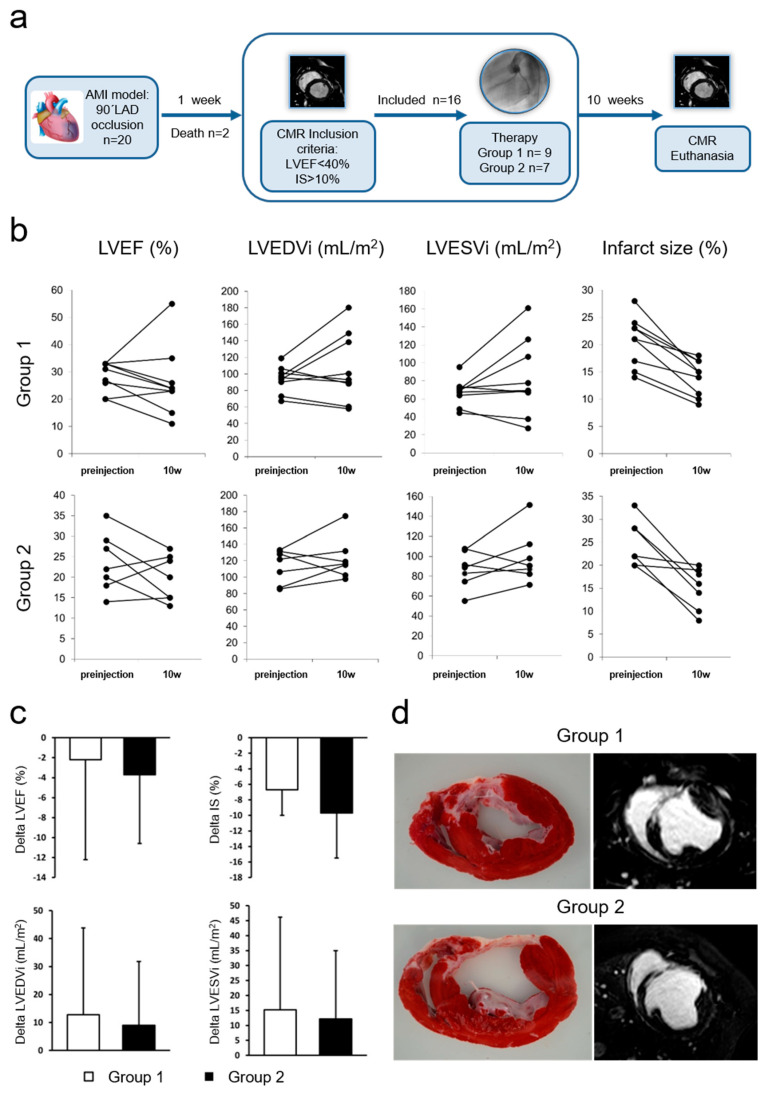
In vivo study in infarcted swine. (**a**) Study design. Experimental workflow illustrating porcine studies. (**b**) Paired data scatterplots showing changes over time in cardiac function parameters as measured with cardiac magnetic resonance (CMR) for the two experimental groups. From left to right, left ventricular ejection fraction (LVEF), left ventricular end diastolic volume indexed to body surface area (LVEDVi), left ventricular end systolic volume indexed to body surface area (LVESVi), and infarct size (IS) in Group 1 (top tier) and Group 2 (lower tier). Group 1 (treatment) = injection of pCPC-HGF-mCherry + pCPC-IGF1-eGFP; Group 2 (control) = injection of pCPC-mCherry + pCPC-eGFP. (**c**) Treatment effects (defined as the difference between pre-injection and 10−week values) from the same parameters. (**d**) Representative CMR and TTC−stained slices from the two studied groups.

Cardiac function parameters, as measured with CMR during the course of the study, are summarized in Figure 6 and Table 2. In the absence of significant differences, cardiac function parameters at 10 weeks were consistently better in Group 1 compared to Group 2 (LVEF was 26 ± 12% vs. 19 ± 5%, LVEDVi was 106 ± 41 mL/m^2^ vs. 122 ± 25 mL/m^2^, LVESVi was 82 ± 42 mL/m^2^ vs. 99 ± 26 mL/m^2^ and IS 14 ± 3% vs. 15 ± 4%, in Group 1 compared to Group 2 animals).

## 4. Discussion

In the present study, we evaluated whether equilibrated overexpression of HGF and IGF growth factors in pCPC could benefit, upon intracoronary transplantation, their therapeutic capacity in a swine model of sub-acute MI. This experimental design allows a similar scenario to the allogeneic approach with hCPC carried out by the CAREMI clinical trial [30,31]. By using cells from the same host specie, a major interference of immunological concerns due to an interspecies transplantation was also avoided. Regarding administration approach, we selected to deliver pCPC one week post-myocardial infarction induction, considering enhanced beneficial effect previously demonstrated after cell differed administration in comparison with the immediate administration [20].

First, we compared pCPC gene expression profile versus hCPC in order to find similarities and differences between porcine and human cells. Although more than 1000 genes were found to be differentially express in pCPC, only 3 (IGF-1, KLF4, and F11R) of the highly preferentially CPC expressed genes that have been validated previously in hCPC were found upregulated. A further analysis of selected markers highly preferentially expressed in hCPC showed a compatible gene expression profile in pCPC, also consistent with previous reported studies in hCPC [37,64]. Therefore, this large difference in pCPC compared with hCPC seems to be probably derived from being obtained from different species.

An extensive analysis of cell surface markers in pCPC populations by flow cytometry demonstrated a typical CPC immunophenotype consistent with previous reported studies in hCPC. We further analyzed by RT-qPCR the expression of F11R and CACNG7, which have been proposed as plausible markers for hCPC. Both F11R and CACNG7 are significantly overexpressed in expanded hCPC compared with MSC and whole human cardiac tissue [64]. Expression of both markers were also confirmed in early stages of hCPC isolation [64]. F11R is a ligand for the integrin LFA1 involved in leukocyte transmigration [66] and is a platelet receptor [67]. In skin, it promotes wound healing by enhancing both homing and secretory activities of MSC [68], regulating also human epidermal stem cell proliferation [69]. Interestingly, it has been also demonstrated that F11R-A2 interactions regulate hematopoietic stem cell fate through Notch signaling [70]. On the other hand, CACNG7 (Calcium Voltage-Gated Channel Auxiliary Subunit Gamma 7) regulates the trafficking and gating properties of AMPA-selective glutamate receptors (AMPARs), promoting their targeting to the cell membrane and synapses. Only broad modified gene expression of CACNG7 has been described in brain tumor compared with normal human fetal neural stem cells [71].

In order to validate functional behavior of cells, pCPC response to cardiogenic differentiation was also analyzed. A comparable response to the behavior of murine CPC under the same cardiogenic differentiation conditions was observed [72], thus demonstrating similar expression profile and cardiac differentiation potential between these two cell populations, despite being isolated from different species.

Forced IGF-1 or HGF overexpression in pCPC were obtained by lentiviral transduction, hence maintaining high levels of expression, even in the case that pCPC would engraft and get signaled for cardiac differentiation. In this sense, although basal IGF-1 expression in pCPC was already higher than hCPC (around 6.77 LogFC), we demonstrated in vitro that pCPC-IGF1-eGFP exhibit a substantial enhanced cardiogenic potential compared to pCPC transduced with empty vector (pCPC-eGFP). Thus, based on these results, we expected that pCPC-IGF1-eGFP could have more chances to contribute to an effective repair of MI injury. Overexpression levels of both growth factors (IGF-1 and HGF) were evaluated in pCPC, using paMSC as additional control of overexpression. Results suggested that both IGF1-eGFP^pos^ populations (paMSC and pCPC) overexpressed IGF-1, but HGF-mCherry^pos^ cultures could be counter-selecting high levels of HGF in the two cell types analyzed; alternatively, the stability of the HGF protein could be much lower than IGF-1, thus limiting the conclusion of our experiments on HGF overexpression. In any case, secreted HGF is clearly and specifically detected in the conditioned medium of HGF-mCherry^pos^ cells (Figure S4).

pCPC gene expression profile was also analyzed after IGF-1 or HGF overexpression in both positive cell populations compared with untransduced cells. Overexpression of CD26 and LRRC59 appear to be upregulated in HGF-mCherry^pos^ cells compared with IG1-GFP^pos^ cells. Curiously, overexpression of CD26 and LRRC59 has been associated with human glioma stem cells stemness [73] and with invasion and poor prognosis in lung adenocarcinoma [74], respectively. Interestingly, pCD73 was the only gene found significantly downregulated in both positive cell populations. CD73 is a widely expressed molecule with a relevant expression in BM and in distinct cancer stem cell models. Moreover, CD73 expression is part of the minimal criteria agreement for the definition of any multipotent MSC [75]. It has been recently reported that pre-treatment of mice with CD39 and CD73 inhibitors facilitates the mobilization of different types of BM-residing stem cells, including MSC, by decreasing the extracellular level of adenosine [76]. The upregulation of CD73 is associated with the overproduction of adenosine, reducing or suppressing antitumor immune responses and, therefore, favoring proliferation, angiogenesis, and metastasis. Importantly, CD73 is currently considered a potent target opportunity for cancer immunotherapy that will be evaluated in future trials [77]. However, no direct link between IGF-1 and HGF signaling and regulation of CD73 has been reported yet. Based on these findings observed in most BM cell populations [76], we could speculate that, after engineered-pCPC (IGF-1/HGF) transplantation, a reduction in CD73 expression could result in a lower extracellular levels of adenosine that would modify adhesion to extracellular matrix, mainly, fibronectin. In summary, overexpression of both growth factors moderately affects the basal expression profile of pCPC. These results were comparable with previous research obtained on paMSC [60] that found moderate alterations in the expression (reduced) of FLT1 and VEGFA in engineered-paMSC coexpressing IGF1-eGFP, as well as a mild increase in ACAN (aggrecan), MYH7 (myosin heavy chain 7), and MEF2C (Myocyte Enhancer Factor 2C) and increased HGFL (Hepatocyte Growth Factor-Like Protein) expression levels in engineered-paMSC coexpressing HGF-mCherry [60].

Previous studies demonstrated that LV-dECM maintained the 3D cardiac macro and microstructure, the native vascular network in a perfusable state and architectural integrity. Furthermore, the effect of dECM on hCPC and hMSC cardiac differentiation was also analyzed and compared in parallel in conventional 2D cultures [63]. Thus, based on these previous studies, we used this model as a surrogate for in vivo niche structure to evaluate the potential effect of the sustained expression of IGF-1 or HGF in the eventual differential biological response of engineered-pCPC to a cardiogenic stimulus. Relevant cardiogenic genes were clearly upregulated in 2D-cultured pCPC-IGF1-eGFP compared with pCPC transduced with EV-eGFP. This upregulation was maximum when pCPC-IGF1-eGFP were co-cultured with LV-dECM. Contrarily, significantly poorer results were obtained with pCPC-HGF-mCherry and pCPC-mCherry populations in both culture conditions. These results seem to be consistent with other previously reported in the literature. IGF-1 signaling, in addition to protecting primary cardiomyocytes from apoptosis induced by hypoxia and serum deprivation [47], also promotes mesodermal expansion in ES differentiation procedures, increasing the CPC pool formation [48], and favoring vascular differentiation [78]. Interestingly, IGF-1 has been also critically involved in differentiation of BM-MSC into cardiomyocyte-like cells [79] and in human ES-differentiation of cardiomyocytes primarily activated by IGF-1 receptor [80]. HGF/Met signaling controls the adult heart homeostasis. HGF mainly (1) promotes pro-survival effects in cardiomyocytes and angiogenesis, protecting against ischemia oxidative stress; (2) activates anti-inflammatory and immunomodulatory signals reducing fibrosis; and, finally, (3) stimulates regeneration through activation of CSC/CPC [42,43,44], and inducing proliferation of cardiomyocytes [46]. Scarce information on the effect of HGF on CSC/CPC differentiation is available, but it has been proposed an in vitro activity favoring BM-MSC differentiation into cardiomyocytes, but inhibiting proliferation [81]. In conclusion, the comparison of expression levels of key cardiogenic induced by IGF-1 or HGF overexpression after 3 weeks of culture with LV-dECM demonstrated the limited inductive capacity of HGF, the superior potential of IGF-1 to promote cardiac differentiation, and the synergism of IGF-1 with inductive signals provided by rat LV-dECM for promoting cardiogenesis of pCPC.

In spite of previous promising results indicating a probable cardiac regeneration capacity, we could not demonstrate long-term functional benefits in a large animal model. This finding, known as “translational axis”, in which clinical effectiveness decreases from small to large animals and ultimately to clinical human studies, has been reported before [82]. This, again, underlines the importance of rigorous preclinical experimentation prior to clinical translation, as well as the importance of using clinically relevant animal models and technologies, such as we have used herein, since faulty translation leads to a lack of results, in addition to increased costs in both economical and societal terms.

Based on all published antecedents, as well as our own work, these results were unexpected. Using allogeneic pCPC, and after intracoronary administration one week after acute MI, we confirmed feasibility and safety of the therapeutic scheme, demonstrating a marked structural and functional benefit in pigs [20,21,22]. Furthermore, we also tested the isolated effect of an intracoronary infusion of microencapsulated IGF-1 (5 × 10^6^) in a swine acute MI model. This strategy demonstrated a significant improvement in LVEF correlating with a reduction in myocardial fibrosis. However, no differences either in infarct size or vascular density were found [52]. Finally, intramyocardial treatment of infarcted swine with paMSC engineered to overexpressed IGF-1 and HGF showed a clear reduction in inflammation in some sections analyzed and promoted angiogenic processes in ischemic tissue, although no significant improvements in cardiac function parameters were obtained nor was any relevant synergistic effect found [60]. Thus, compared with paMSC, we considered that pCPC would be more appropriate vehicle for the evaluated strategy. It could be argued that the procedure to obtain pCPC overexpressing both growth factors could alter their biological capacities; however, in vitro functional evaluations and our own experience suggest this is unlikely. Therefore, although it could be considered that the reduced number of animals used in this study could be a major limitation, it is envisioned that probably the in vivo effects provoked by engineered-pCPC in order to ameliorate myocardial ischemia/reperfusion damage in the MI porcine model may be limited.

## 5. Conclusions

We have obtained and characterized engineered pCPC for the overexpression of IGF-1 or HGF, combined with compatible fluorescent markers. Both growth factors were stably expressed, although HGF at a lower level, and none of them altered significantly the basal gene expression profile of pCPC. In addition, pCPC-IGF1-eGFP showed a higher cardiogenic response, evaluated in co-cultures with decellularized scaffolds, compared with native pCPC and pCPC-HFG-mCherry cells. Finally, intracoronary co-administration of pCPC-IGF1-eGFP and pCPC-HFG-mCherry were evaluated in vivo in sub-acute infarcted pigs, 1 week after LAD occlusion; no statistically significant functional improvement was found.

## Figures and Tables

**Table 1:** Main pCPC DEG previously identified in hCPC as differentially overexpressed in comparison with BM−MSC and HDF.

Protein	Description	Others	Type	Log FC pCPC/hCPC	RU pCPC
Membrane					
F11R	Junctional adhesion molecule A	CD321/JAM1	Int M	2.45	16.3
IL1R1	Interleukin 1 receptor, type I	IL-1R-alpha	TK-R	1.07	182
IGF2R	IGF Cation-independent mannose-6-phosphate receptor	CD222/M6P-R	Mb-R	0.31	16.1
DPP4	Dipeptidyl peptidase 4	CD26	Int M Gly	−0.38	190.2
CACNG7	Calcium Voltage-Gated Channel Auxiliary Subunit Gamma 7	TARP Gamma-7	TM	−0.98	42.5
ECE1	Endothelin-Converting Enzyme 1	ECE	Enz	−1.6	157
GPR4	G Protein-Coupled Receptor 4	G-PCR 19	TM	−7.74	0.21
Secretome					
IL1B	Interleukin 1 Beta	IL-1 Beta	Cyt	9.1	100.4
IGF1	Insulin-Like Growth Factor 1	-	GF	8.77	55
IL1A	Interleukin 1 Alpha	Hematopoietin-1	Cyt	−1.81	122
TGF b1	Transforming Growth Factor Beta 1	TGF-Beta-1	GF	−2.76	1565
Cytoplasm					
PAPSS2	3′-Phosphoadenosine 5′-Phosphosulfate Synthase 2	Adeno 5-Phosphosulfate Kinase	kin	2.77	1889
P4HA1	Prolyl 4-Hydroxylase Subunit Alpha 1	P4HA	Hydrox	1.22	1423
PHD1	Prolyl Hydroxylase Domain-Containing Protein 1	EGLN2	Hydrox	−0.42	81.2
Nuclear					
IGF2BP2	Insulin-Like Growth Factor 2 MRNA Binding Protein 2	IMP2	RNA-BP	0.05	175.8
IGF2BP3	IGF2 MRNA-Binding Protein 3	IMP3	RNA-BP	0.42	71.4
GATA4	GATA Binding Protein 4	-	TF	−0.3	44
WT1	Wilms Tumor 1	WR33	TF	−0.91	6.29

Log FC = log fold change; RU = relative units; Int M = integral membrane; TK-R = tyrosine kinase receptor; Mb-R = membrane-bound receptor; Int M Gly = integral membrane glycoprotein; TM = transmembrane; Enz = enzyme; Cyt = cytokine; GF = growth factor; Kin = kinase; Hidrox = hydroxilase; RNA-BP = RNA binding protein; TF = transcription factor.

**Table 2:** Main cardiac parameters calculated from CMR exams performed throughout the study.

Groups	Group 1 (Treatment, *n* = 9)		Group 2 (Control, *n* = 7)	
	1 Week (Preinjection)	10 Weeks	1 Week (Preinjection)	10 Weeks
LVEF (%)	28 ± 5	26 ± 12	23 ± 7	19 ± 5
LVEDVi (mL/m^2^)	93 ± 15	106 ± 41	113 ± 20	122 ± 25
LVESVi (mL/m^2^)	67 ± 15	82 ± 42	86 ± 18	99 ± 26
Infarct Size (%)	20 ± 4	14 ± 3	24 ± 4	15 ± 4
Δ LVEF (%)	n/a	−2.2 ± 10	n/a	−3.7 ± 6.9
Δ LVEDVi (mL/m^2^)	n/a	12.8 ± 31	n/a	9 ± 22.8
Δ LVESVi (mL/m^2^)	n/a	15.2 ± 30	n/a	12.2 ± 21.2
Δ Infarct Size (%)	n/a	−6.7 ± 3.3	n/a	−9.7 ± 5.8

Data presented as mean ± standard deviation; LVEF: Left ventricular ejection fraction; LVEDVi: Left ventricular end diastolic volume indexed to body surface area; LVESVi: Left ventricular end systolic volume indexed to body surface area; Infarct area is expressed as % of the left ventricle; n/a: not applicable. Group 1 (treatment) = injection of pCPC-HGF-mCherry + pCPC-IGF1-eGFP; Group 2 (control) = injection of pCPC-mCherry + pCPC-eGFP.

## Data Availability

The data behind the conclusions of this study are available from the corresponding author upon reasonable request. All transcriptomic data derived from this study are deposited in the Gene Expression Omnibus (GEO) database, and are accessible through the accession number GSE84070.

## Supplementary Materials

The following are available online at https://www.mdpi.com/article/10.3390/cells10102571/s1, Table S1: Antibodies used in flow cytometry, western blot and immunofluorescence (IF) assays; Table S2: Primer sequences used in quantitative real-time PCR (RT-qPCR) experiments; Table S3: Complete list of differentially expressed genes (DEG) in pCPC, BM-MSC, and HDF obtained by RNA-Seq; Table S4. Complete list of DEG in pCPC compared with hCPC; Table S5: Comparative surface markers expression levels on CPC/CSC from pig, human, and mouse/rat; Table S6: Surface markers expression in independent isolates of pCPC (*n* = 4, a–d) and hCPC (*n* = 2, hCPC3 and hCPC4); Table S7: Statistical analysis of gene expression data from Figure 5e; Figure S1: Schematic summary of (a) LV-dECM obtention from rat heart and (b) co-culture strategy; Figure S2: Comparison of gene expression levels by RT-qPCR of IGF-1, HGF, and their main receptors (IGF-1R and Met) in engineered-pCPC populations and hCPC; Figure S3: Evaluation of HGF and IGF-1 expression by western blot and characterization of HGF/mCherry cell response to co-culture with decellularized rat left ventricle (LV-dECM) scaffolds; Figure S4: ELISA protein quantification of HGF and IGF-1 in conditioned media of pCPC transduced populations compared with hCPC.
