# Supplementary material for: Intracoronary Delivery of Porcine Cardiac Progenitor Cells Overexpressing IGF-1 and HGF in a Pig Model of Sub-Acute Myocardial Infarction
Source: Cells. 2021 Sep 28;10(10):2571. doi: 10.3390/cells10102571 (PMC8534140; doi:10.3390/cells10102571)
Supplement: Supplementary file 1 [file cells-10-02571-s001.zip › cells-1314684-supplementary.v3.pdf]

Supplementary Methods

RNA-Seq analysis

Sequenced reads were quality-controlled and pre-processed using Cutadapt v1.6 [1] to remove adaptor contaminants, as described previously [2]. Resulting reads were aligned and gene expression quantified using RSEM v1.1.19 [3] over human reference GRCh37 and Ensembl genebuild 65. Only genes with at least 1 count per million in at least 3 samples were considered for statistical analysis. Data were then normalized and differential expression tested using the Bioconductor package EdgeR v3.0.8 [4]. We considered as differentially expressed those genes with a Benjamini-Hochberg adjusted p value ≤ 0.05. For the set of differentially expressed genes, functional analysis was performed using the topGO v2.10 Bioconductor R package [5] with annotations from org.Hs.eg.db and GO.db v2.8. For functional analysis, the genes at the extracellular space according to its GO annotations were used. Enrichment was performed using the full list of equally localized genes as reference. Top biological processes and molecular functions were selected using the Weighted Fisher method implemented by topGO with $p<0.01$. To visualize relationships between genes and biological processes, a chord plot was generated using the R visualization package GOPlot [6].

Plasmid construction

Human HGF was amplified from pBABE-puro TPR-HGF plasmid (from Bob Weinberg's lab, Addgene plasmid #10901) using paired primers: 5′-CAGCTATCTAGAATGTGGGTGACCAAACTC-3′ and 5′-CAGCTACCCGGGTCATAGTATGTCAGCGCAT-3′; then it was cloned in the pRRLsin18.CMV.IRES.mCherry vector using XbaI/XmaI polylinker restriction sites to obtain the pRRLsin18.CMV.HGF.IRES.mCherry lentiviral vector. Rat IGF-1 was obtained from pExpress-IGF1 (Clon Image ref.7300903, Gene service) using specific primers: 5′-CAGCTAGGATCCATGTCGTCTTCACATCTC-3′ and 5′-CAGC-TACCCGGGTCATAGTATGT CAGCGCAT-3′; then the insert was cloned in the Bam-HI-XmaI unique restriction sites of donor pRRLsin18.CMV.IRES.eGFP lentiviral shuttle vector to obtain the pRRLsin18.CMV.IGF1.IRES.eGFP vector. Both lentiviral vectors, pRRLsin18.CMV.HGF.IRES.mCherry and pRRLsin18.CMV.IGF1.IRES.eGFP, were functionally validated by immunofluorescence (IF) and western blot by transfection in HEK293T cells with lipofectamine 2000 (ThermoFisher Scientific), following the provider instructions.

Sequence alignments of rat IGF-1 and human HGF translated proteins were confirmed to be 93.5% and 93.3% identical with respective porcine proteins (AAH86374.2 versus NP_999421.1, and AAG53460.1 versus XP_013835241.2, respectively), with few non-conservative amino acids.

Lentiviral particle production

Viral particles were produced by transient plasmid transfection into HEK293T cells grown in DMEM-high glucose, 10% heat-inactivated FBS, 2 mM L-glutamine, 50 U/mL penicillin/streptomycin, and 2 mM Hepes (all reagents from Sigma-Aldrich). The day before transfection, $1x10^6$ trypsinized cells were seeded on poly-D-lysine (Sigma-Aldrich) treated wells from six-well plates. Each different lentiviral transfer vector (10 μg DNA) was mixed with lentiviral helper plasmids (pRSV-Rev and pMDLg/pRRE packaging vectors; and pMD2.VSVG envelope-encoding vector) in an equimolar ratio using the calcium-phosphate co-precipitation

method. The following day, the transfection solution was removed, the cells were rinsed with 1X PBS, and medium without FBS was added to the cells. Viral supernatants were harvested at 48 h post-transfection, cleared by low-speed centrifugation, and filtered through a 0.45-μm low-protein-binding filter (Corning). Viral stocks were concentrated by ultracentrifugation in a SW28 Beckman rotor at 90000 g (26000 rpm) for 2 h, at 4°C. Pellets containing lentiviral particles were air-dried and resuspended O/N at 4°C in 400-600 μL of media. Viral titres (transducing units; TU/mL) were calculated by FACS analysis on transduced HEK293T cells and particles were quantified by RT-qPCR on supernatants (particles/mL). Values obtained were around 10^7-10^8 TU/mL and in a 1:100 TU/particles ratio.

ELISA

For quantification of secreted HGF and IGF-1, we assayed the conditioned medium generated by two hCPC isolates or the different engineered pCPC batches, cultured during 48 h in serum-free medium. We employed a specific ELISA, for the quantification of human IGF-1 (*R&D Systems; Cat. Num: DG100B*) or rat HFG (*R&D Systems; Cat. Num: MHG00*) following the manufacturer instructions.

Supplementary Tables and Figures

Table S1. Antibodies used in flow cytometry, western blot and immunofluorescence (IF) assays.

Antibody	Catalog number (Company)
anti-CD11R3 (FITC)-mouse anti-pig	MA5-28279 (Thermo Fisher Scientific)
anti-CD15 (PE)-mouse anti-human	555402 (BD Pharmingen)
anti-CD29 (FITC)-mouse anti-human	ab21845 (Abcam)
anti-CD31 (FITC)-mouse anti-human	555445 (BD Pharmingen)
anti-CD31 (FITC)-mouse anti-pig	MCA1746F (ABD Serotec)
anti-CD34 (PE)-mouse anti-human	555822 (BD Pharmingen)
anti-CD44 (FITC)-rat anti-human	ab19622 (Abcam)
anti-CD45 (PE-Cy7)-mouse anti-human	557748 (BD Pharmingen)
anti-CD45 (FITC)-mouse anti-pig	MCA1222F (ABD Serotec)
anti-CD49f (FITC)-rat-anti-human	555735 (BD Pharmingen)
anti-CD73 (PE)-mouse anti-human	550257 (BD Pharmingen)
anti-CD90 (FITC)-mouse anti-human	555595 (BD Pharmingen)
anti-CD105 (FITC)-mouse anti-pig	ab53318 (Abcam)
anti-c-kit (CD117) human (APC)-mouse anti-human	550412 (BD Pharmingen)
anti-c-kit (CD117) porcine (PE)-rabbit anti-human	A4502 (DakoCytomation)
anti-CD166 (PE)-mouse anti-human	559263 (BD Pharmingen)
anti-tubulin (HRP)-goat anti-mouse	P0447 (DAKO)
anti-GFP-rabbit polyclonal	ab290 (Abcam)
anti-IGF1 (H-70)-rabbit anti-human	sc-9013 (Santa Cruz Biotech)
anti-HGF (H-145)-rabbit polyclonal	sc-7949 (Santa Cruz Biotech)
mouse IgG1 (FITC)-isotype control	MG101 (Caltag Laboratories)
mouse IgG2a (FITC)-isotype control	ab1281 (Abcam)
mouse IgM-λ-isotype control	550963 (BD Pharmingen)
rat IgG2b (FITC)-isotype control	ab37364 (Abcam)
rat IgM (FITC)-isotype control	553408 (BD Pharmingen)
anti-rabbit (Alexa 488)-goat anti-rabbit	A11034 (Invitrogen)
anti-rabbit-(HRP)-goat anti-rabbit	P0448 (DAKO)
anti-rabbit (Biotin)-goat anti-rabbit	ab6720 (Abcam)
anti-rabbit (Alexa 568)-donkey anti-rabbit	A10042 (Invitrogen)
Streptavidin (Cy3)	43-4315 (Molecular Probes)
Streptavidin (FITC)	43-4311 (Molecular Probes)

APC = allophycocyanin; Cy3 = cyanine 3; HRP = horseradish peroxidase; FITC = fluorescein isothiocyanate; PE = phycoerythrin.

Table S2. Primer sequences used in quantitative real-time PCR (RT-qPCR) experiments.

Gene	Forward (5′-3′)	Reverse (5′-3′)
36B4	TCATCCAGCAGGTGTTTGAC	CAGACATACGCTGGCAACAT
ACT4	GGGAATGGGACAAAAAGACA	CATCCCAGTTGGTGATGATG
ACTB	CCCCTGCAGTTCGCCATGGAT	CACCATCACGCCCTGGTGTCG
ACTC1	CTCCTTTGTCACCACTGCTGAGCG	AGCAGCTGTAGCCATCTCATTCTCA
Bmi1	ATGCTGCCAATGGCTCTAAT	CCTGTTCTGGTCAAAGAACTCA
CACNG7	TAAAGAACCAAGCCCACCAC	TCAGCCTCTTCCTCGTGTTC
CD9	GAGGCACCAAGTGCATCAA	AGCCATAGTCCAATGGCAAG
CD26 (porcine)	GGACTCTCAGCCCAAACGCCA	GAGCCCTCCGGATCCACTGC
CD29 (porcine)	GCGTCGCCGAGTCTCCTCCT	GACTCCCGCTCGGCCTGTCC
CD44 (porcine)	TCAACAGCACGCTGCCCACC	GCATTGGGGTGGATCCGGGG
CD49 (porcine)	GGCAGGCAGGCTGGTGACAG	GCCTGGAGAGGGGACCCTGG
CD73 (porcine)	CACAGCCGCCTGGAGCAGAC	AGCAGCAGCACGTGGGGTTC
CD98 (porcine)	GGCACCGACTCCTCCGACCT	AAGCTCCAGCTGCACCAGCG
CD166 (porcine)	ACCCCTTGAAGAAGCGGTGGTCA	TCACAGAGCAGGTGAATGGCATTTGT
CKIT	TTCACAGAGACTTGGCGGCCA	CGGGTAGCCGAGCGTTTCCTT
CX3CR1	CACTCACCATGTCCACCATC	GGCCAAAGGCAAAAATAAGG
CXCL12	GTGTGTCAGGCCTCCGTCCG	CCGGTTTCTCATCGCTGAGGCA
FGFR2	AAACACGTGGAAAAGAACGG	TCACATTGAACAGAGCCAGC
FLK1	CAAAACTGTCGTGATTCCATGTC	TTCTGTTACCATCAGGAACAAACCT
F11R	TCGAGAGGAAACTGTTGTGC	GAAGAAAAGCCCGAGTAGGC
F11R (porcine)	TCTTGTGCTCCCTGACGTTG	AATTTCCACTCCACACGGGG
GAPDH	TGGAAGGACTCATGACCACA	AGCACCAGTAGAAGCAGGGA
GAPDH (porcine)	AACTGCTTGGCACCCCTGGC	CTGGAGAGCCCCTCGGCCAT
GATA4	TGGCCGCCAACCACGGC	GCGTGGGCACGTAGACGGG
GUSB (porcine)	CCCCAGCGATGGACCCAGGA	TCGGCCTCGAAGGGGAGGTG
HGF	TCCTAAGAAGCCGAGAGGCA	AGCAGACATGGTCTTCCACC
HGF (rat)	GCAGACACCACACCGGCACA	ATGGCCTCGGCTTGCCATCG
IGF1 (porcine)	GACGCTCTTCAGTTCGTGTG	CTCCAGCCTCCTCAGATCAC
IGF-1R (porcine)	CAGTCCTAGCACCTCCAAGC	GTCTTCGGCCACCATACAGT
IGF2	TCAGGCTAGTCTCTCCTCGG	TTGAGGGGTTCAATTTTTGG
IGF2R	GAAGGTGAAGGTCGGAGT	GAAGATGGTGATGGGATTTC
IGFBP2	GCCCTCTGGAGCACCTCTACT	CATCTTGCACTGTTTGAGGTTGTAC
Klf4	GCGAACCCACACAGGTGAGAAA	AATGCCCGGTCGCACTTCTGG
LRRC59	GGCAGCGGCGGCTGGAAATA	TCGCTTGGCCGCTTTGAGGG
MET (porcine)	CCCAATTTCTGACTGAGGGA	TAGGACCACCAGTGGAGACC
MLC2V	GAAACTTAAGGGGGCAGACC	CCTCCTTGGAAAACCTCTCC
MYH7	GCTCTCAGGTCCCTGCCAGCTTG	GCCTCCCCAAATGCGGCCATC
NKX2.5	AAGTGCGCGCCCTCCTTCTCA	AGCGCGCACAGCTCTTTCTTATC
NRP1	ACCCGGAGAGAGCCACCCAC	CTGTGGCAGCTGGCCTGGTC
MOYF	CTTTCGCCGCAGACGCTGGA	GGTGGTGGCGCTGTGCTTCT
SOD1	TCCATGTCCATCAGTTTGGA	AGTCACATTGCCCAGGTCTC

SOD2	CTTCGTCTTCCTCCTCGTTG	AAACCTATGTGGGTTGCTCG
SOX2	AGCGCATGGACAGCTACGCG	CTGCATCTGAGCCGCGCTGT
TNNI3	CCAACTACCGCGCCTACGCC	CCGCTCCTCTGCCTCCCGTT
VEGFA	ATCTTCAAGCCGTCCTGTGT	TCTCTCCTATGTGCTGGCCT

Table S3. Complete list of differentially expressed genes (DEG) in pCPC, compared with BM-MSC and HDF obtained by RNA-Seq.

Table S4. Complete list of DEG in pCPC compared with hCPC. The highest overexpressed genes in pCPC compared with hCPC (n=3 isolates for each cell type) are indicated in green. Those underexpressed in pCPC are indicated in red. The list has been organized according to the level of differential expression and a color code has been included, accordingly.

Table S5. Comparative surface markers expression levels on CPC/CSC from pig, human and mouse/rat. Expression analysis in porcine (pCPC, n=4 isolates) and human (hCPC, n=2 isolates) cells was carried out by flow cytometry. The intensity of the expression and the numbers in parenthesis correspond to the percentage of cells that express the indicated surface protein in a representative isolate for each CSC/CPC population.

**Protein**	**pCPC**	**hCPC**	**Mouse/rat CSC/CPC (*)**	**Other names**
**CD15**	m-H (40)	m-H (40)	n.d.	**SSEA-1**
**CD44**	vH (20)	m-H (20)	vH (80)	**HCAM**
**CD49f**	vH (20)	vH (80)	vH (100)	**ITGA6**
**CD73**	vH (20)	m-H (100)	n.d.	**NTSE**
**CD90**	vH (80)	vH (100)	vH (100)	**Thy1**
**CD166**	vH (100)	m (60)	vH (80)	**ALCAM**
**CD117**	m (40)	m (40)	m (40)	**KIT/SCFR**
**CD31**	null	null	l (40)	**PECAM-1**
**CD34**	null	null	null	**-**
**CD45**	null	null	null	**PTPRC**

(*) Data for mouse/rat CSC/CPC extracted from the literature.
vH = very high; m-H = medium-high; m = medium; l = low; null = no expression; and n.d. = not determined.

Table S6. Surface markers expression in independent isolates of pCPC (n=4, a-d) and hCPC (n=2, hCPC3 and hCPC4). Numbers correspond to the percentage of positive cells for each surface protein in the indicated CPC population.

	**pCPC**				**hCPC**	
	**a**	**b**	**c**	**d**	**hCPC3**	**hCPC4**
**CD29**	98.0	98.9	100.0	96.0	96.3	96.0
**CD44**	94.4	94.7	100.0	93.2	94.2	98.3
**CD90**	97.9	94.8	98.3	89.5	78.7	75.3
**CD105**	84.9	91.8	76.2	63.4	79.5	93.4
**CD11R3**	≤2	≤2	≤2	≤2	≤2	≤2
**CD31**	≤2	≤2	≤2	≤2	≤2	≤2
**CD45**	≤2	2.6	≤2	≤2	≤2	n.d.

n.d. = not detected

Table S7. Statistical analysis of gene expression data from **Figure 5E**.

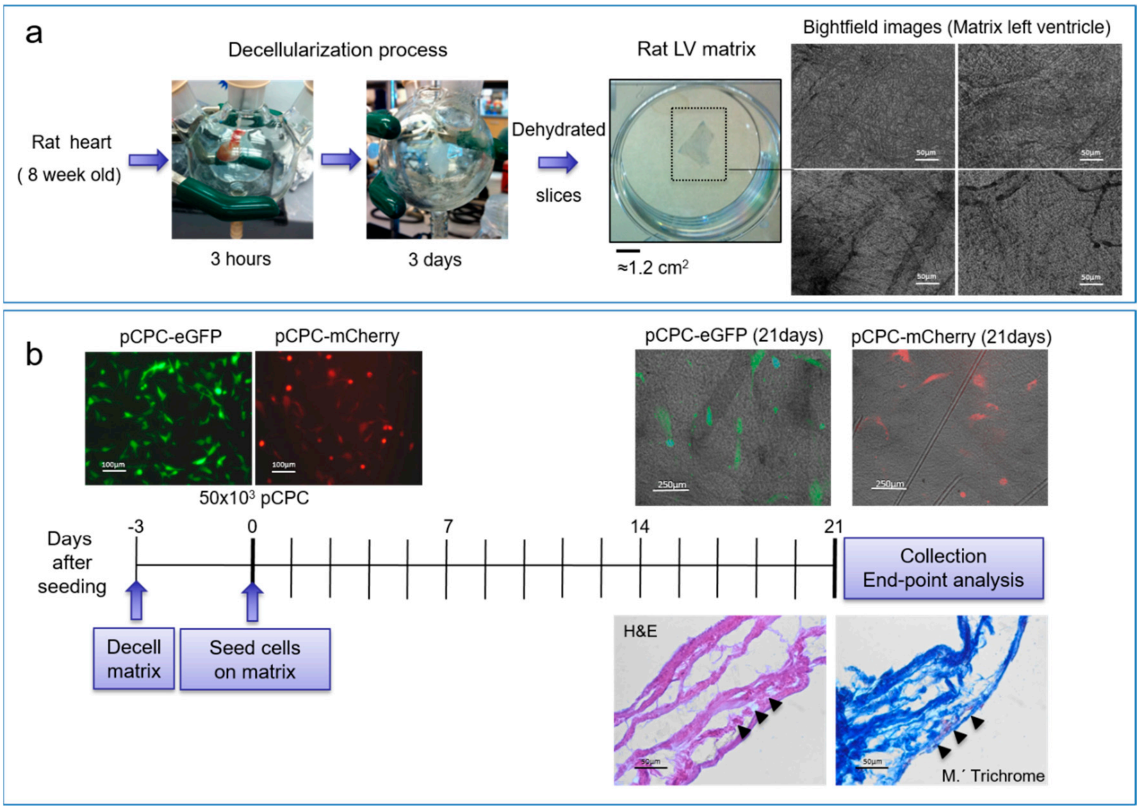

Figure S1. Schematic summary of (**a**) LV-dECM obtaining from rat heart and (**b**) co-culture strategy.

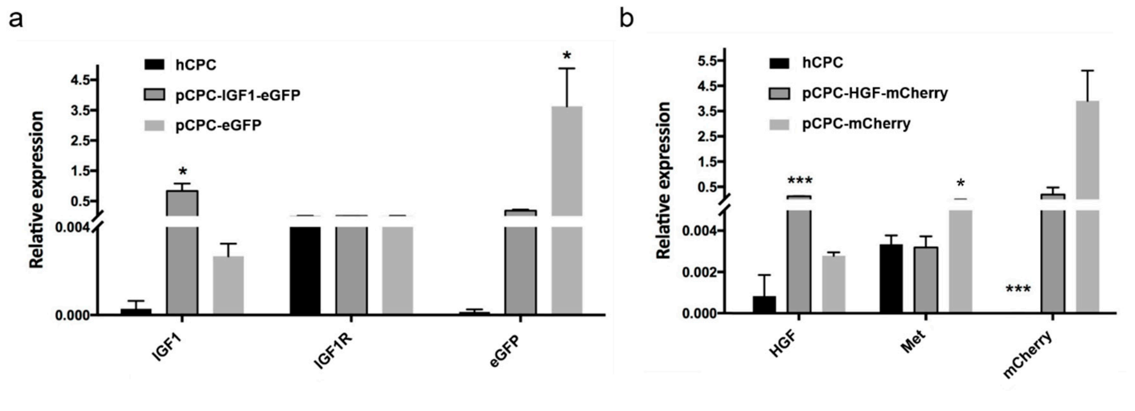

Figure S2. Comparison of gene expression levels by RT-qPCR of IGF-1, HGF, and their main receptors (IGF1R and Met) in engineered-pCPC populations and hCPC . Levels of (**a**) IGF1, IGF1R and eGFP and (**b**) HGF, Met and mCherry are shown. Human or porcine primers were tested for hCPC and pCPC amplifications, respectively. In the case of HGF, primers pair designed for rat origin were used (see Table S2 for more details). GAPDH was used as housekeeping gene (n=2). For IGF1: *$p<0.05$, pCPC-IGF1-eGFP vs. hCPC; for eGFP: *$p<0.05$, pCPC-eGFP vs. hCPC; for HGF: ***$p<0.001$, pCPC-HGF-mCherry vs. hCPC; for Met: *$p<0.05$, pCPC-mCherry vs. hCPC; and for mCherry: ***$p<0.001$ hCPC vs pCPC-mCherry.

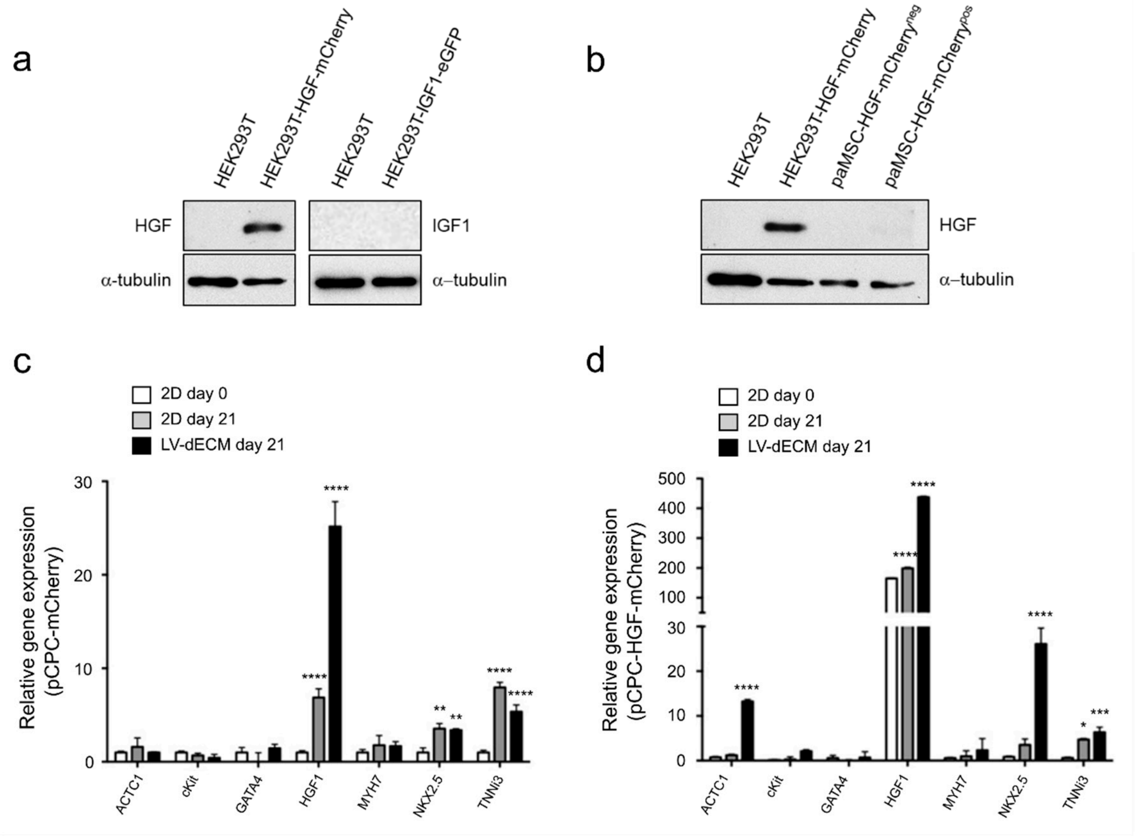

Figure S3. Evaluation of HGF and IGF-1 expression by western blot and characterization of HGF/mCherry cell response to co-culture with decellularized rat left ventricle (LV-dECM) scaffolds. (**a**) HEK 293T cells transfected with pRRLsin18.CMV-HGF-IRES-mCherry or pRRLsin18.CMV-IGF1-IRES-eGFP were compared by western blot for the expression of HGF and IGF-1, respectively. (**b**) Compared HGF expression levels, by western blot, in HEK 293T cells transfected with pRRLsin18.CMV-HGF-IRES-mCherry and their basal control HEK 293T, with pCPC transduced with pRRLsin18.CMV-HGF-IRES-mCherry (pCPC-HGF-mCherry) and their negative control cells (pCPC-mCherry). α-tubulin was used for loading control. (**c,d**) Comparative cardiogenic gene expression of pCPC-HGF-mCherry and pCPC-mCherry co-cultured with LV-dECM scaffolds for 21 days. (**c**) Control cells (transduced with empty vector pCPC-mCherry), were culture in conventional 2D culture or on rat LV-dECM scaffolds for 21 days. For HGF1: ****$p \leq 0.0001$, pCPC-mCherry 2D day 21 vs. pCPC-mCherry 2D day 0 and pCPC-mCherry dECM day 21 vs. pCPC-mCherry 2D day 0. (**d**) pCPC-HGF-mCherry cells were culture in conventional 2D culture or on rat LV-dECM scaffolds for 21 days. Expression of the indicated cardiogenic genes in both pCPC populations was evaluated by RT-qPCR (day 21) and compared with their corresponding basal expression at day 0. For HGF1: ****$p<0.0001$, pCPC-HGF-mCherry 2D day 21 vs. pCPC-HGF-mCherry 2D day 0 and pCPC-HGF-mCherry dECM day 21 vs. pCPC-HGF-mCherry 2D day 0; for NKX2.5: ****$p<0.0001$, pCPC-HGF-mCherry dECM day 21 vs. pCPC-HGF-mCherry 2D day 0; for TNNi3: *$p<0.05$ in pCPC-HGF-mCherry 2D day 21 vs. pCPC-HGF-mCherry 2D day 0; ***$p<0.001$, pCPC-HGF-mCherry dECM day 21 vs. pCPC-HGF-mCherry 2D day 0. In scaffold assays GAPDH was used as housekeeping gene (n=3).

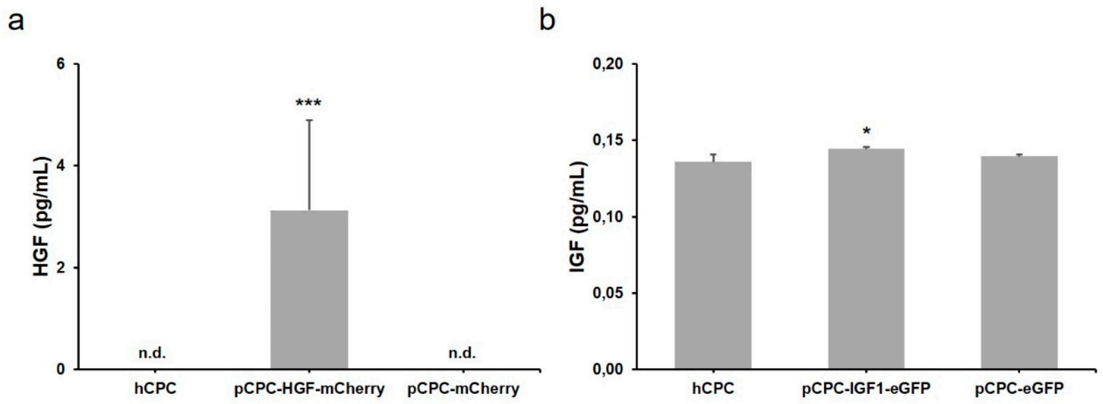

Figure S4. ELISA protein quantification of HGF and IGF-1 in conditioned media of pCPC transduced populations compared with hCPC. (**a**) Detection of HGF levels secreted by pCPC-HGF-mCherry and pCPC-mCherry in comparison with hCPC (n=2-4). ***$p<0.001$ vs. hCPC; n.d.= not detected. (**b**) IGF-1 levels secreted by pCPC-IGF1-eGFP and pCPC-eGFP versus hCPC (n=2-4). *$p<0.05$ vs. hCPC.

Supplementary References

1. Martin, M. Cutadapt removes adapter sequences from high-throughput sequencing reads. *EMBnet.journal* **2011**, *17*, doi:10.14806/ej.17.1.200.
2. Nakazato, T.; Ohta, T.; Bono, H. Experimental Design-Based Functional Mining and Characterization of High-Throughput Sequencing Data in the Sequence Read Archive. *PLoS One* **2013**, *8*, doi:10.1371/journal.pone.0077910.
3. Li, B.; Dewey, C.N. RSEM: Accurate transcript quantification from RNA-Seq data with or without a reference genome. *BMC Bioinformatics* **2011**, *12*, doi:10.1186/1471-2105-12-323.
4. Robinson Mark, D.; McCarthy Davis, J.; Smyth Gordon, K. edgeR: a Bioconductor package for differential expression analysis of digital gene expression data. *Bioinformatics* **2010**, *26*.
5. Alexa, A.; Rahnenfuhrer, J. topGO: topGO: Enrichment analysis for Gene Ontology. R package version 2.18.0. *R Top. Doc.* **2010**.
6. Walter, W.; Sánchez-Cabo, F.; Ricote, M. GOplot: An R package for visually combining expression data with functional analysis. *Bioinformatics* **2015**, *31*, 2912–2914, doi:10.1093/bioinformatics/btv300.
